# Control of mitotic chromosome condensation by the fission yeast transcription factor Zas1

**DOI:** 10.1083/jcb.201711097

**Published:** 2018-07-02

**Authors:** Christoph Schiklenk, Boryana Petrova, Marc Kschonsak, Markus Hassler, Carlo Klein, Toby J. Gibson, Christian H. Haering

**Affiliations:** 1 Cell Biology and Biophysics Unit, European Molecular Biology Laboratory, Heidelberg, Germany; 2 Structural and Computational Biology Unit, European Molecular Biology Laboratory, Heidelberg, Germany

## Abstract

Although the formation of rod-shaped chromosomes is vital for the correct segregation of eukaryotic genomes during cell divisions, the molecular mechanisms that control the chromosome condensation process have remained largely unknown. Here, we identify the C_2_H_2_ zinc-finger transcription factor Zas1 as a key regulator of mitotic condensation dynamics in a quantitative live-cell microscopy screen of the fission yeast *Schizosaccharomyces pombe*. By binding to specific DNA target sequences in their promoter regions, Zas1 controls expression of the Cnd1 subunit of the condensin protein complex and several other target genes, whose combined misregulation in *zas1* mutants results in defects in chromosome condensation and segregation. Genetic and biochemical analysis reveals an evolutionarily conserved transactivation domain motif in Zas1 that is pivotal to its function in gene regulation. Our results suggest that this motif, together with the Zas1 C-terminal helical domain to which it binds, creates a cis/trans switch module for transcriptional regulation of genes that control chromosome condensation.

## Abstract (precis)

How chromosomes compact into rod-shaped structures is a longstanding unresolved question of cell biology. Schiklenk et al. identify the transcription factor Zas1 as a central regulator of mitotic chromosome condensation in fission yeast and show that it uses a conserved transactivation domain–based mechanism to control gene expression.

## Graphical Abstract

**Figure:**
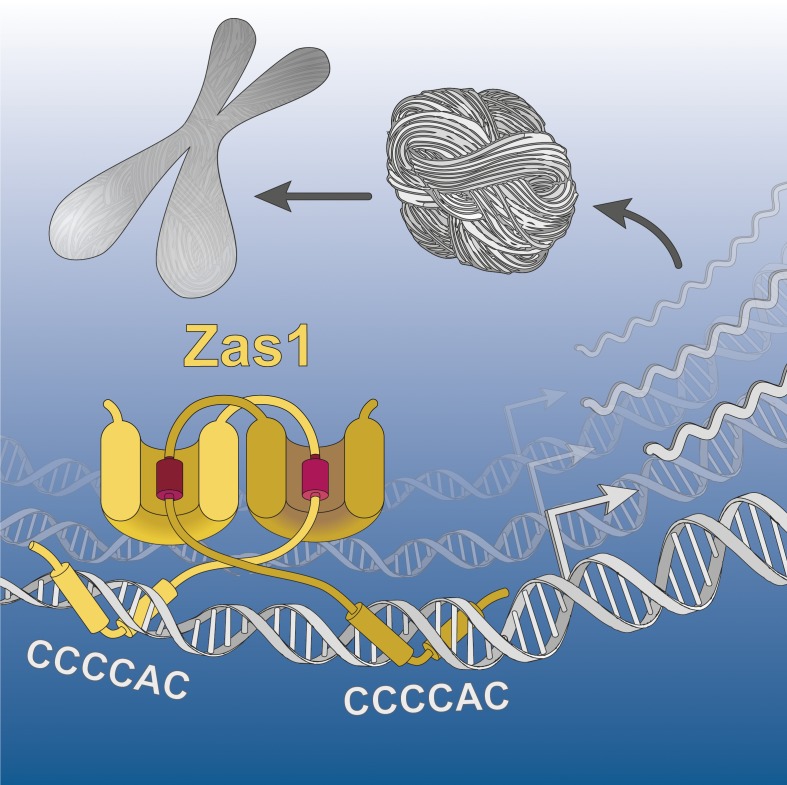

## Introduction

The reshaping of interphase chromatin into rod-shaped mitotic and meiotic chromosomes is one of the most remarkable events in the eukaryotic cell cycle. Even though the phenomenology of the chromosome condensation process had been well described by the end of the 19th century (Flemming, 1882), our understanding of the molecular mechanisms that drive the massive changes in chromatin organization has remained surprisingly limited (Moser and Swedlow, 2011). This is largely because, despite the identification by proteomic studies of thousands of proteins that associate with mitotic chromosomes (Ohta et al., 2010), only very few of these proteins seem to have clearly defined roles in the formation of mitotic chromosomes.

One of the major nonhistone constituents of mitotic chromosomes are multi-subunit protein complexes named condensins (Hirano et al., 1997; Sutani et al., 1999). Their Smc2 and Smc4 subunits are members of the ubiquitous Structural Maintenance of Chromosomes protein family and form, together with the Cnd2 kleisin subunit, a large ring-shaped protein complex that is thought to topologically encircle chromatin fibers (Anderson et al., 2002; Onn et al., 2007; Cuylen et al., 2011). The kleisin subunit binds to two additional subunits that are named Cnd1 and Cnd3 in fission yeast and composed of α-helical repeat motifs (HEAT repeats). Whereas the Cnd3 subunit functions in the recruitment of condensin complexes to chromosomes by creating a DNA binding site together with Cnd2 (Kschonsak et al., 2017), the function of Cnd1 has remained unknown.

Depletion, inactivation, and knockout experiments demonstrated that condensin complexes are crucial for the correct formation of mitotic chromatids in *Xenopus laevis* egg extracts as well as human cultured cells or of meiotic bivalents in mouse oocytes, respectively (Ono et al., 2003; Lee et al., 2011; Houlard et al., 2015). Topoisomerase II (topo II), another abundant component of mitotic chromosomes, is similarly essential during the formation of discrete chromatids by disentangling catenated sister DNAs (Uemura et al., 1987; Adachi et al., 1991) in a process that is directed by the action of condensin complexes (Sen et al., 2016; Piskadlo et al., 2017). The recent discoveries that a limited set of proteins—including histones, histone chaperones, condensin, and topo II—are sufficient for the assembly of chromatid-like structures from in vitro reconstituted chromatin substrates (Shintomi et al., 2015) and that nucleosome assembly is not strictly required for this process (Shintomi et al., 2017) highlight the central importance of condensin and topo II in the formation of mitotic chromosomes.

How condensin is activated to ensure the timely formation of mitotic chromosomes during cell division and is prevented from compacting chromosomes during interphase are key questions that have largely remained unanswered. Activation of the complex through phosphorylation by mitotic kinases and control of its access to chromosomes are thought to be the main regulatory pathways, although the identity of the activating kinases, their target sites in the condensin complex, and the mechanisms to restrain nuclear and/or chromosomal localization of condensin appear to vary considerably between different species (Bazile et al., 2010; Piazza et al., 2013). Little is known about the control of condensin activity at the level of gene regulation. In budding yeast, down-regulation of the expression of the Cnd3/Ycg1 HEAT-repeat subunit, in combination with constitutive proteasomal degradation of the protein, limits condensin binding to interphase chromosomes (Doughty et al., 2016). Whether a similar control of condensin activity exists in other species is unclear. The findings that overexpression of a condensin kleisin subunit in *Drosophila melanogaster* results in large-scale nuclear rearrangements (Bauer et al., 2012) and that overexpression of the human Cnd3/NCAPG/G2 condensin subunits correlates with the tumorigenic potential of certain cancer cells (Liu et al., 2017; Zhan et al., 2017; Zhang et al., 2018) emphasize, however, the importance of a strict cellular control of condensin expression.

Insights into the mechanisms that regulate condensin levels will require the identification of gene-specific transcription factors (TFs) that control the expression of condensin subunits. Most eukaryotic TFs are characterized by the presence of DNA-binding domains that bind to the promoters of target genes and of regions of intrinsic disorder that function as transactivation domains (TADs) to recruit transcriptional coactivators (Staby et al., 2017). This recruitment depends on the interaction of conserved short linear motifs at the core of TADs with globular domains in the binding partners. Prominent examples are the binding of the TAD motifs of the *Saccharomyces cerevisiae* TFs Gal4 and Gcn4 to a hydrophobic groove in the mediator subunit Gal11/Med15 (Piskacek et al., 2016) or the association of a TAD motif of the cell cycle checkpoint regulator E2F with a pocket formed between tandem cyclin-fold domains of the retinoblastoma tumor suppressor protein Rb (Lee et al., 2002). Whether there exist TFs that use these or similar principles to control the transcription of genes involved in chromosome condensation is not yet known.

Here, we use a live-cell microscopy–based assay in the fission yeast *Schizosaccharomyces pombe*, which generates a quantitative time-resolved measure of the degree of chromosome compaction as cells pass through mitosis (Petrova et al., 2013; Schiklenk et al., 2017), to screen a collection of mutant strains for regulators of mitotic chromosome condensation. We identify a C_2_H_2_ zinc-finger (ZF) TF named Zas1 as a key regulator for the expression of the condensin subunit Cnd1, as well as for severalother genes with roles during cell division and additional cellular pathways. Genetic and biochemical analysis reveals a conserved TAD motif, which is essential for the role of Zas1 in gene regulation and interacts with a large helical domain located in the C-terminal region of the protein to potentially create a cis/trans regulatory switch. Our findings reveal the molecular mechanism of transcriptional control of condensin and have implications for the evolution of transcriptional mechanisms that regulate key events of the eukaryotic cell cycle.

## Results

### Mutations in the *zas1* gene slow down chromosome condensation dynamics

**Figure 1.**
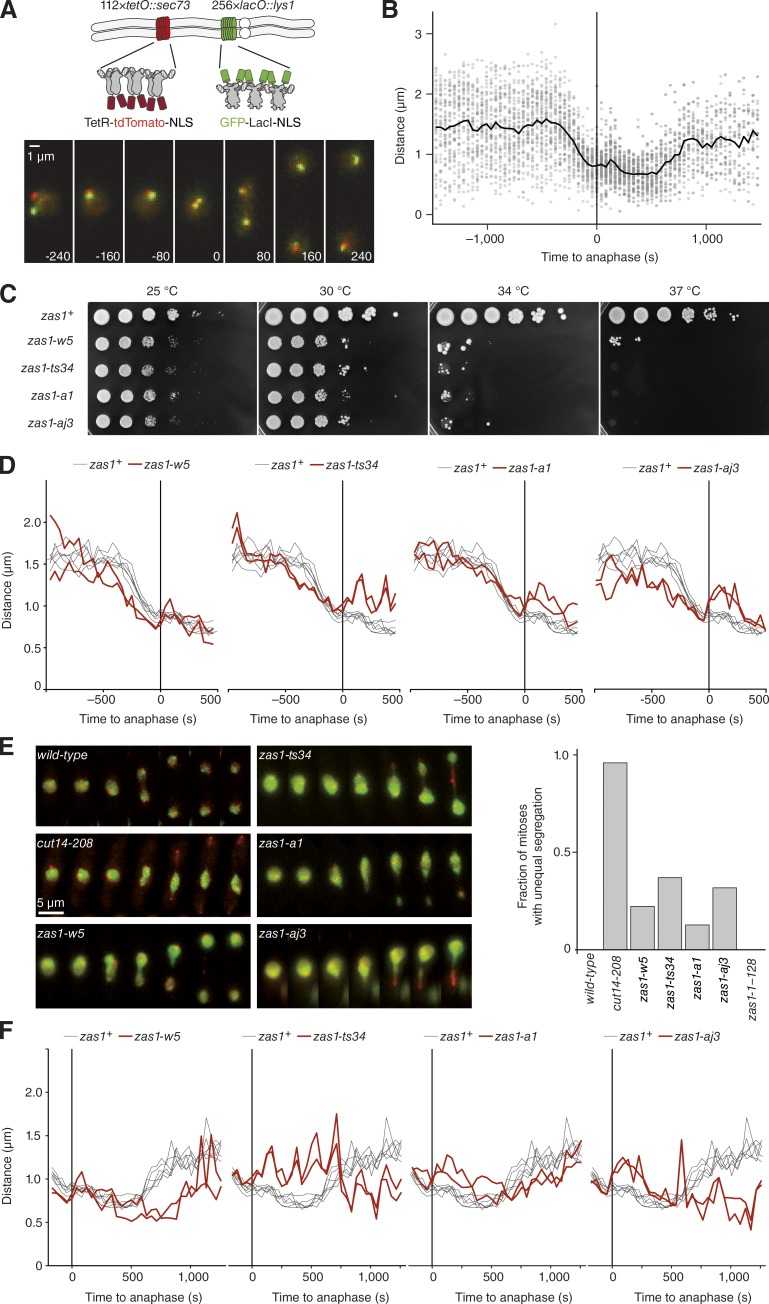
**Chromosome condensation and segregation defects in *zas1* mutants. (A)** The quantitative chromosome condensation assay tracks the 3D positions of two loci on the longest fission yeast chromosome arm, which are fluorescently labeled by the introduction of *lac* and *tet* operator repeats and expression of GFP–LacI and TetR–tdTomato fusion proteins (FROS), as cells pass through mitosis (strain C2926). Bar, 1 µm. **(B)** Taking the mean of single-cell measurements (gray circles) from a group of cells after temporal alignment to anaphase onset (*t* = 0, frame of first sister FROS splitting) results in a condensation curve (black line). **(C)** Cells of wild-type *zas1^+^* (C1283) or *zas1* mutant strains (C3693 [*zas1-w5*], C3717 [*zas1-ts34*], C4506 [*zas1-a1*], C3673 [*zas1-aj3*]) were grown at 25°C in liquid culture to logarithmic phase, spotted in a 10-fold dilution series onto rich medium plates, and incubated for 5 d at the indicated temperatures. **(D)** Condensation curves of *zas1* mutants (red lines, C3766 [*zas1-w5*], C3809 [*zas1-ts34*], C3399 [*zas1-a1*], C4106 [*zas1-aj3*]; *n* = 2 independent experiments) and wild-type *zas1^+^* control strains (black lines; strain C2926; *n* = 6 independent experiments). **(E)** Example time series of unequal chromosome segregation events in *cut14* and *zas1* ts mutant strains with FROS labels. The signal of the green channel was enhanced to visualize nuclear morphology. Quantitation of the fraction of mitotic cells that display unequal segregation at 34°C. Strains as in C and C3005 (*cut14-208*) and C4049 (*zas1-1–289*). *n* = 592 (wild-type), *n* = 121 (*cut14-208*), *n* = 59 (*zas1-w5*), *n* = 60 (*zas1-ts34*), *n* = 127 (*zas1-a1*), *n* = 54 (*zas1-aj3*), and *n* = 187 (*zas1-1–289*) mitoses scored. **(F)** Decondensation curves of *zas1* mutants as in D.

To search for factors that control chromosome condensation dynamics, we screened datasets of ∼1,100 randomly generated temperature-sensitive (ts) fission yeast strains for mutants that displayed alterations in the dynamics of chromosome condensation (Petrova et al., 2013). This dataset was generated by integrating two spectrally distinct fluorescent repressor-operator systems (FROSs) into defined positions within the longest *S. pombe* chromosome arm and recording the 3D Euclidean distance between the two FROS foci using live-cell imaging as cells pass through mitosis (Fig. 1 A and Video 1; Schiklenk et al., 2017). In the resulting time series, we defined the time point at which we detected first FROS foci splitting as anaphase onset. Alignment of the distance time series of many mitoses allowed us to calculate mean distances for each time point and thereby generate condensation curves that quantitatively describe the chromosome condensation process (Fig. 1 B).

Using this assay, we identified three independent ts mutant strains that displayed notably slowed condensation rates. For each of these strains, we mapped the condensation phenotype to a distinct single mutation in the *zas1* gene locus using a whole-genome sequencing approach (Petrova et al., 2013). We then tested whether mutations in the *zas1* locus were responsible for the observed changes in condensation rates by reintroducing each mutation (*zas1-w5*, *zas-a1*, and *zas1-aj3*) into an otherwise wild-type genetic background. The three resulting *zas1* mutant strains showed reduced growth at 25°C and, as expected, were unable to grow at a restrictive temperature of 34°C (Fig. 1 C). Importantly, two of the three mutant strains (*zas1-w5* and *zas1-aj3*) showed notably decreased condensation rates when measured at the restrictive temperature (Fig. 1 D). Condensation rates were furthermore decreased in a previously described *zas1* ts mutant (*zas1-ts34*; Fig. 1 D; Okazaki and Niwa, 2000). The fact that another mutant version of *zas1*, which had been isolated independently of our microscopy screen, equally affects condensation dynamics strongly supports the notion that the *zas1* gene locus plays a key role in controlling the timing of mitotic chromosome assembly.

Chromosome condensation defects regularly result in the missegregation of sister chromatids during anaphase. Although the fraction of cells with segregation defects in *zas1* mutant strains was lower than in cells with a ts mutation in the condensin subunit gene *cut14*, we frequently observed that the bulk of the chromosome mass failed to segregate equally into the daughter cells in all *zas1* mutants (Fig. 1 E). This was even the case for the *zas1-a1* mutation, which had a milder effect on chromosome condensation dynamics than the other three mutations (Fig. 1 D).

Whereas the massive chromosome segregation defects in the *zas1-ts34* and *zas1-aj3* mutants impeded analysis of chromosome decondensation after the completion of anaphase, we were able to assess decondensation rates in *zas1-w5* and *zas1-a1* cells. Compared with *zas1^+^* wild-type cells, decondensation took longer in *zas1-a1* and was strongly delayed in *zas1-w5* mutant cells (Fig. 1 F). We conclude that a function encoded by the *zas1* gene locus is essential for the timely condensation of chromosomes upon entry into mitosis, correct chromatid segregation during anaphase, and rapid decondensation after the completion of anaphase.

### The Zas1 protein controls the timing of chromosome condensation

**Figure 2.**
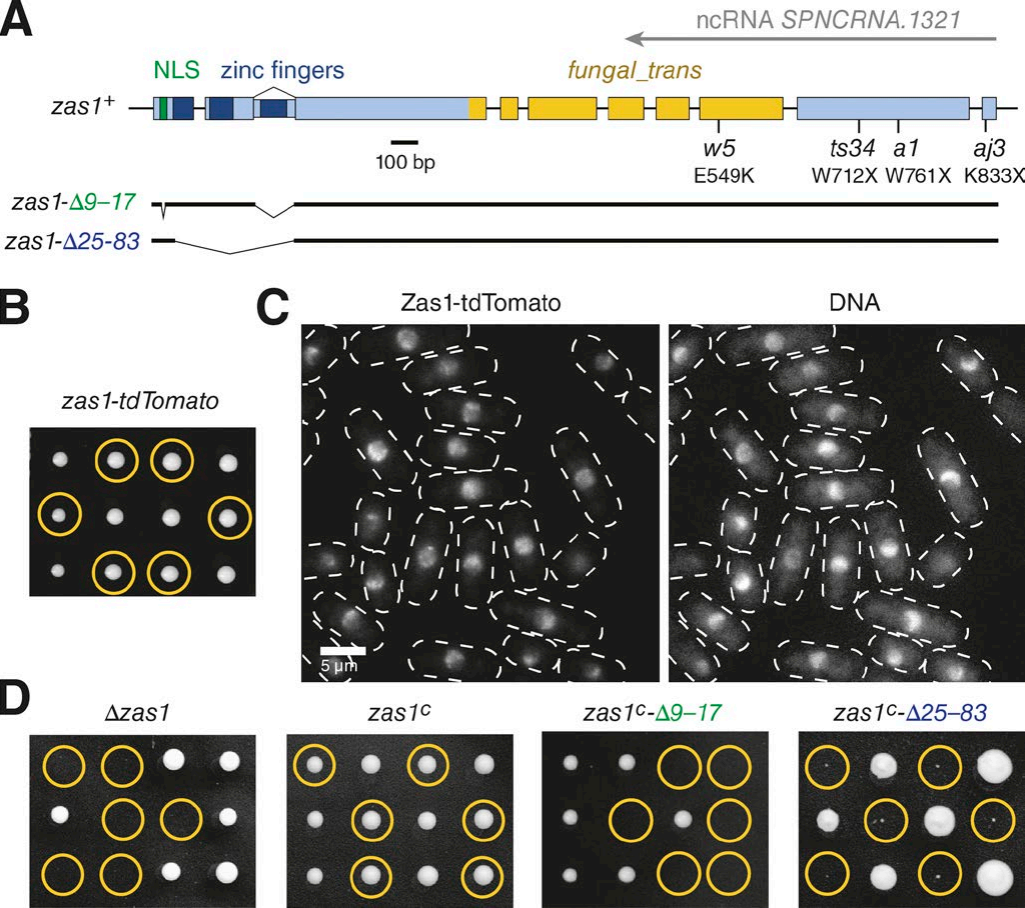
**Zas1 localization to chromosomes is essential for its function. (A)** Schematic representation of the *zas1* gene locus. The positions of a NLS (green), of the C_2_H_2_ ZF DNA binding motifs (dark blue), of a predicted fungal TF domain (*fungal_trans*, yellow), of a noncoding antisense RNA transcript, and of ts mutations and their effect on the Zas1 protein (with X representing a stop codon) are indicated. Lines represent noncoding introns. **(B)** Tetrad analysis of a heterozygous *zas1-tdTomato*/*zas1^+^* diploid fission yeast strain (C5091) after 5 d at 25°C. Circles identify the position of spores bearing the *zas1-tdTomato* gene. **(C)** Example images of Zas1-tdTomato localization (left) and chromatin staining with Hoechst (right; strain C3782). **(D)** Tetrad analysis of heterozygous *Δzas1*/*zas1^+^*, *zas1^c^*/*zas1^+^*, *zas1^c^-Δ9–17*/*zas1^+^*, and *zas1c-Δ25–83/zas1^+^* diploid fission yeast strains (C4005, C4083, C4470, and C4093) after 5 d at 25°C. Circles identify the position of spores bearing the *Δzas1*, *zas1^c^*, *zas1^c^-Δ9–17*, or *zas1c-Δ25–83* genes, respectively.

The *zas1* gene had been reported to be one of only a few alternatively spliced ORFs in the *S. pombe* genome and to encode proteins of 845 or 897 amino acids in length, respectively (Okazaki and Niwa, 2000). Both proteins contain a nuclear localization sequence (NLS) and, depending on the splice isoform, two or three C_2_H_2_ ZF DNA binding domains (InterPro IPR013087) close to their N termini. The C-terminal region of the Zas1 protein shares weak sequence homology with domains found in fungal TFs (InterPro IPR007219, Pfam PF04082: *fungal_trans*). Furthermore, the 3′ region of the *zas1* ORF is transcribed in the antisense direction to produce a long noncoding RNA (ncRNA *SPNCRNA.1321*; Rhind et al., 2011). Notably, all ts *zas1* mutations are located within this part of the gene (Fig. 2 A).

To exclude the possibility that the condensation defects in the *zas1* mutants were caused by effects on the noncoding antisense transcript, we inserted 2.9 kb of DNA encoding two tandem copies of the red fluorescent protein Tomato (tdTomato) at the 3′ end of the *zas1* ORF, which effectively disrupted the ncRNA promoter. Similar to the deletion of the entire antisense transcript region (see below), disruption of the ncRNA promoter had no apparent effect on cell viability or chromosome segregation, which rules out an essential role of the antisense transcript (Fig. 2 B). Hence, the product of the *zas1* gene locus that is pivotal for chromosome condensation must be the Zas1 protein.

### Zas1 localization to chromosomes is essential for its function

We used the tdTomato fusion construct to monitor the cellular localization of Zas1. Live-cell microscopy revealed that Zas1-tdTomato was highly enriched in the cell nucleus at all stages of the cell cycle and remained associated with chromosomes throughout mitosis (Fig. 2 C and Video 2). Segmentation based on confocal microscopy images of GFP-tagged histone H3 allowed us to estimate that approximately one-third of the nuclear pool of Zas1 localizes to the nucleolus (Fig. S1 A), which occupies a roughly equivalent part of the nuclear volume (Sawin and Nurse, 1996). Although Zas1 is clearly able to enter the nucleolus, it is not enriched in this subcompartment. The pattern of Zas1 localization within the chromatin region appeared nonhomogeneous, which indicates that the protein localizes to distinct sites of the fission yeast genome.

If Zas1 needed to associate with chromosomes to perform its essential role, we reasoned that its NLS and ZF DNA binding domains would be indispensable for cell viability. We first generated a cDNA version of the *zas1* ORF that codes for the 845-residue protein with two ZFs (*zas1^c^*). Replacement of the endogenous *zas1* ORF with this cDNA version did not affect cell proliferation, which is consistent with a previous study (Fig. 2 D; Okazaki and Niwa, 2000). Versions of this cDNA construct in which we had deleted either the NLS (*zas1^c^-Δ9–17*) or the two ZFs (*zas1^c^-Δ25–83*) were, in contrast, unable to sustain cell growth (Fig. 2 D). The localization of Zas1 to the nucleus and its binding to DNA via its ZFs are therefore essential aspects of Zas1 function.

### Genome-wide mapping identifies Zas1 binding sites in promoter regions

To map the sites of Zas1 enrichment in the fission yeast genome, we performed chromatin immunoprecipitation (ChIP), followed by next-generation sequencing (seq), of a version of Zas1 tagged with a PK_6_ epitope (Fig. S1 B). Consistent with the observed localization of Zas1 to chromosomes, we obtained ∼5-fold more DNA from the Zas1-PK_6_ immunoprecipitation than from the untagged control (Fig. S1 C). Comparison of the ChIP-seq reads from the Zas1-PK_6_ strain to those from an untagged control strain allowed us to unambiguously identify bona fide Zas1 binding sites. We aligned sequencing reads from two independent replicates to the fission yeast genome and used model-based analysis of ChIP-seq data to identify peaks of enriched binding (Zhang et al., 2008; Feng et al., 2011).

**Figure 3.**
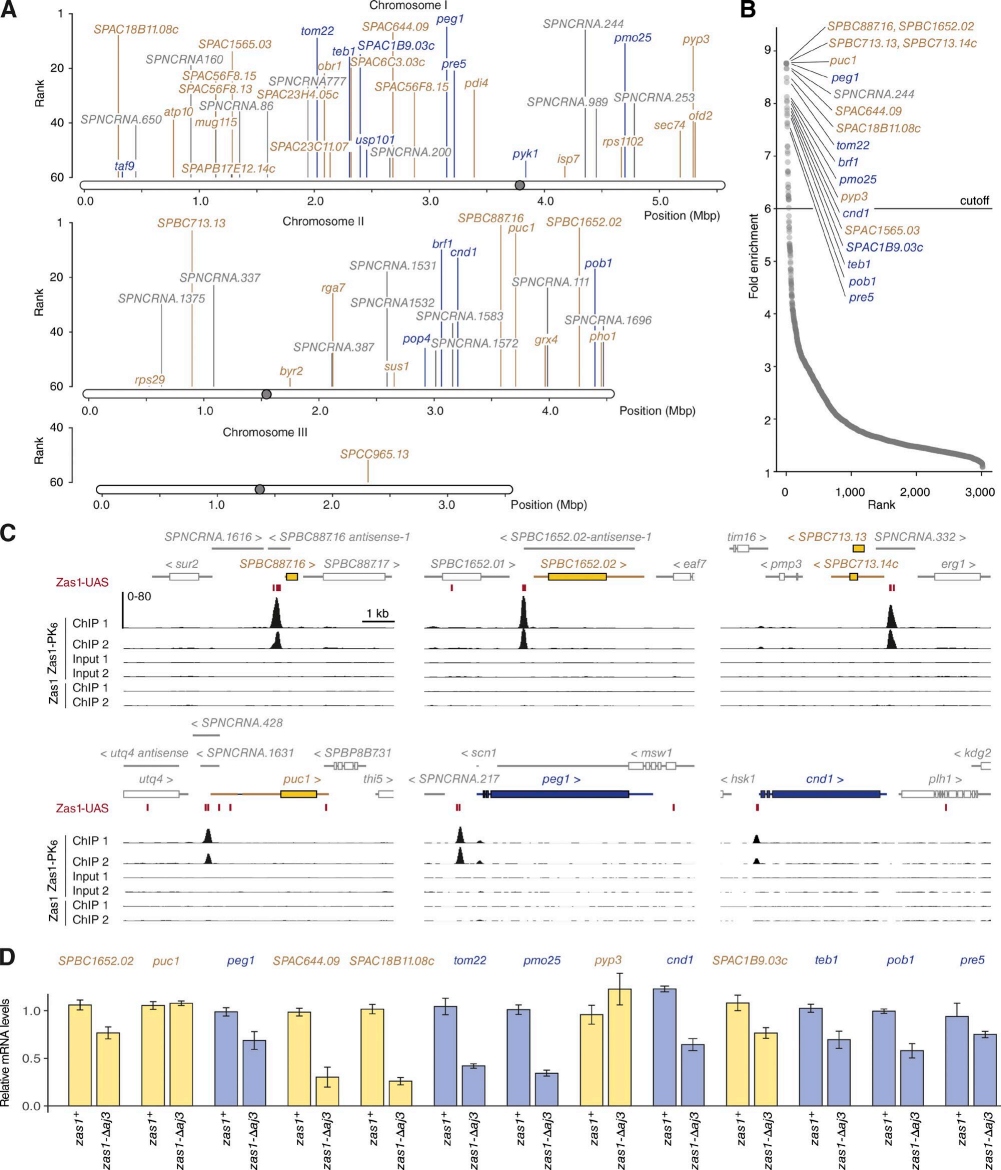
**Genome-wide mapping of Zas1-binding sites. (A)** Genomic positions of the 60 highest-scoring Zas1-binding sites identified by ChIP-seq. **(B)** Enrichment score plot for 3,017 sites in the fission yeast genome identified by Zas1-PK_6_ ChIP-seq. Essential gene names are shown in dark blue, nonessential gene names in yellow, and ncRNA in gray. **(C)** ChIP-seq profiles from two independent experiments of a strain expressing Zas1-PK_6_ (C4120) or an untagged control strain (C28) at the top five binding loci (*SPBC887.16*, *SPC1652.02*, *SPBC713.13*/*SPBC713.14c*, *puc1*, and *peg1*) and the condensin subunit gene *cnd1*. The y-axis indicates the number of reads per 20-bp bin. **(D)** Relative mRNA levels in *zas1^+^* wild-type and *zas1-Δaj3* mutant strains (obtained from tetrad dissection of a *zas1-Δaj3/zas1^+^* strain, C4718) measured by RT-qPCR (mean ± SD; *n* = 8 replicates from four independent tetrads).

The majority of Zas1 binding peaks were distributed along fission yeast chromosomes I and II (Fig. 3 A). Remarkably, 31 of the 40 top-ranked peaks coincided with promoter regions of coding or noncoding genes (Fig. 3 B and Table S1), consistent with the hypothesis that Zas1 functions as a gene-specific TF. At the highest-scoring binding sites, which include the promoters of the uncharacterized ORFs *SPBC887.16*, *SPBC713.13*, and *SPBC713.14c*, as well as the cyclin gene *puc1* and the microtubule regulator gene *peg1*, Zas1 binding was restricted to a narrow peak (Fig. 3 C). The list of top-ranked peaks also included the promoter region of the *cnd1* gene, which encodes one of the two HEAT-repeat subunits of the condensin protein complex (Fig. 3 C). We also inspected the genes encoding the other four subunits of condensin and the gene encoding topo II, but could not detect an enrichment of Zas1 binding at their promoter regions (Fig. S1 D). Likewise, the ribosomal DNA repeats located in the vicinity of both telomeres of chromosome III did not contain distinct Zas1 binding peaks (Fig. S1 E), which is consistent with the finding that Zas1 is not specifically enriched in the nucleolus (Fig. S1 A).

### Zas1 is a gene-specific TF

To test whether Zas1 controls the expression of the genes it binds to, we compared the transcript levels of 13 genes that displayed a prominent Zas1-binding peak in their promoter regions between *zas1^+^* cells and cells in which we had deleted the part of the *zas1* gene that encodes the last 12 amino acid residues to generate an allele that corresponds to the *aj3* nonsense point mutation (*zas1-Δaj3*). The majority of these genes (11/13) were down-regulated by 20–75% in *zas1-Δaj3* cells, and a single gene (*pyp3*) was up-regulated (Fig. 3 D). Although transcript levels of the gene that encodes the cyclin-fold protein Puc1 were unaltered, Puc1 protein levels were clearly reduced in the mutant (Fig. S2 A).

We performed gene ontology term analysis to identify any potential underlying commonalities of the genes regulated by Zas1. To this end, we listed all 31 genes for which the ChIP-seq analysis showed an enrichment of Zas1 binding in the promoter region by a factor of 6.0 or higher (Fig. 3 B) and used the online tool AnGeLi (Bitton et al., 2015) to compare gene ontology term frequencies in the Zas1 gene list to those of all protein-coding genes in the *S. pombe* genome. This analysis revealed that genes involved in cellular component organization (56.5% in Zas1 targets, 27.0% in all genes, P < 0.003) and mitotic nuclear division (17.4% in Zas1 targets, 3.8% in all genes, P < 0.010) were overrepresented in the Zas1 target gene list. We conclude that Zas1 functions as a TF for a specific set of genes, including genes that play roles during cell divisions.

### Zas1 recognizes a specific upstream activator sequence

**Figure 4.**
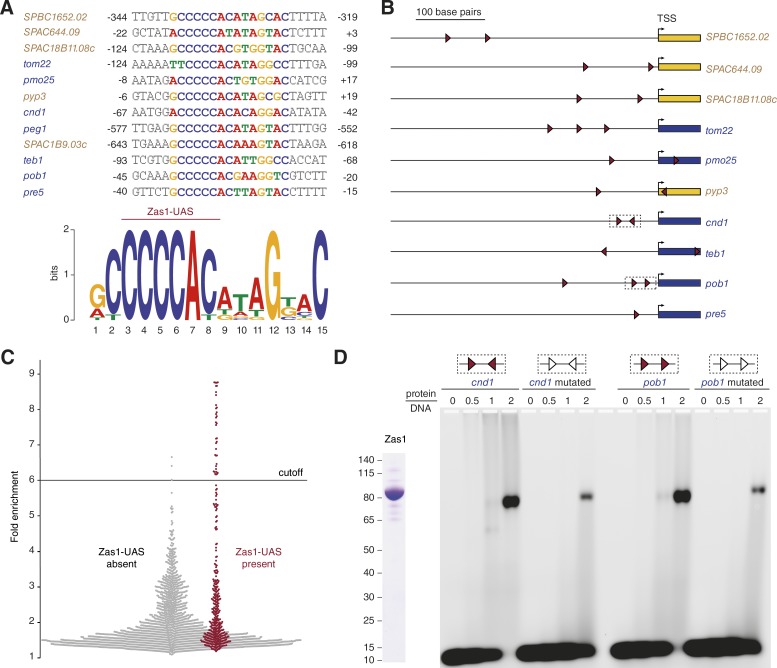
**Zas1 binds the DNA sequence motif CCCCAY. (A)** Sequence alignment of the Zas1-UAS motifs identified within the promoter sequences with highest ChIP-seq peaks. Numbers indicate base pairs relative to the transcription start site. **(B)** Positions of the Zas1-UAS 5′-CCCCAY-3′ (right red arrow) and its reverse complement sequence (left red arrow) of genes controlled by Zas1. **(C)** Zas1 ChIP-seq enrichment scores of peak regions with (red) or without (gray) Zas1-UAS sequences. **(D)** EMSA of recombinantly purified full-length Zas1 protein and 39-bp dsDNA substrates that contain unmodified (red) or mutated (white) Zas1-UAS motifs from the *cnd1* or *pob1* promoter sequences. Numbers above the lanes indicate the molar ratio of Zas1 protein to DNA. SDS-PAGE shows the purified Zas1 protein after staining with Coomassie blue.

Taking into consideration that ZFs bind specific DNA sequences, we used the sequences underlying the ChIP peaks at the promoters of the 12 genes that we had shown to be up- or down-regulated in the *zas1-Δaj3* mutant (Fig. 3 D) to query the motif prediction servers MEME and DMINDA2 (Bailey and Elkan, 1994; Yang et al., 2017). Both servers identified the sequence 5′-CCCCAY-3′ (C, cytosine; A, adenine; Y, pyrimidine) or the reverse complement sequence 5′-RTGGGG-3′ (R, purine; T, thymine; G, guanine) as a consensus sequence motif, which we refer to as Zas1-bound upstream activating sequence (Zas1-UAS; Fig. 4 A). Furthermore, a prediction of the DNA binding sites based on the amino acid sequences of the Zas1 ZFs (Persikov and Singh, 2014) returned the Zas1-UAS as top result for all promoter sequences that we analyzed (not depicted). Notably, most of the analyzed promoter sequences contain two or more instances of the Zas1-UAS (Fig. 4 B), and at least one of them overlaps with the Zas1 ChIP-seq peak in the vicinity of the transcription start sites (Fig. 3 C).

We then asked whether the Zas1-UAS we identified from the subset of Zas1 ChIP-seq peaks was also common to the sequences underlying the other Zas1 ChIP-seq peaks. Remarkably, we found at least one Zas1-UAS consensus sequence in 37 of the 40 Zas1 binding sites that were enriched 6.0-fold or higher in the ChIP-seq dataset (Fig. 4 C). These data strongly suggest that the Zas1-UAS is the core DNA binding sequence of Zas1. To challenge this hypothesis, we tested in an electrophoretic mobility shift assay (EMSA) binding of purified Zas1 to two double-stranded 39-bp DNA substrates from the *cnd1* and *pob1* promoters, which each contain two copies of the Zas1-UAS. Whereas Zas1 was able to shift both substrate DNAs in this assay, binding was notably reduced when we introduced point mutations into the Zas1-UAS (Fig. 4 D).

### Zas1 controls expression of the condensin subunit Cnd1

**Figure 5.**
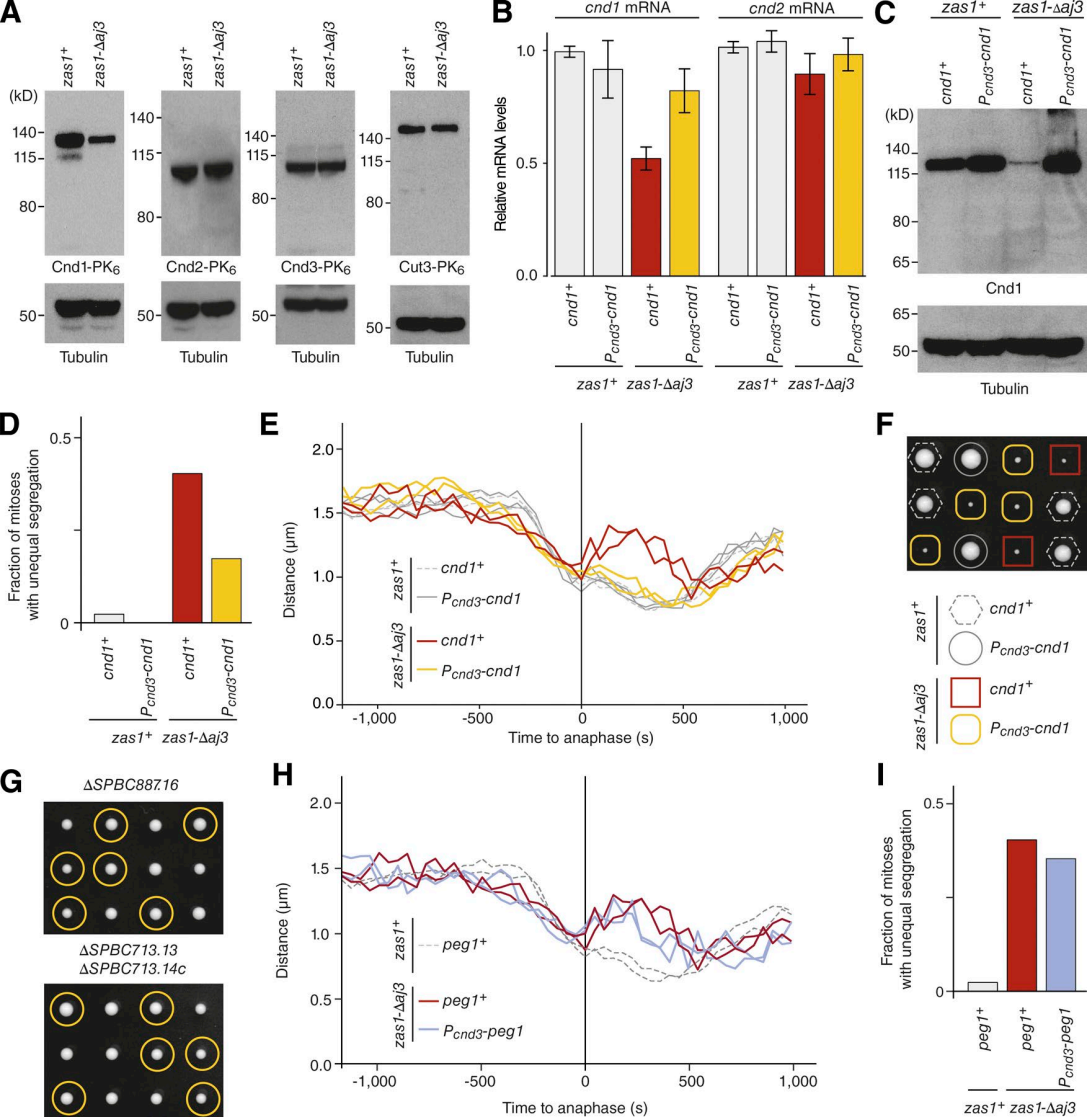
**Zas1-dependent control of Cnd1 or Peg1 expression is not solely responsible for condensation defects. (A)** Comparison of protein levels by immunoblotting of whole-cell extracts from asynchronous cultures of strains derived from dissection of the same *zas1^+^*/*zas1-Δaj3* tetrad (C4543, C4610, C4870, C4990) against the PK_6_ tag fused to condensin subunits Cnd1, Cnd2, Cnd3, or Cut3. Immunoblotting against α-tubulin serves as loading control. **(B)** RT-qPCR signals for *cnd1* and *cnd2* transcripts in asynchronous *zas1^+^* or *zas1-Δaj3* mutant cells expressing *cnd1* from either its endogenous promoter (*cnd1^+^*) or the *cnd3* promoter (*P_cnd3_-cnd1*). Graphs show mean ± SD of measurements from three independent tetrads (C4718). **(C)** Comparison of Cnd1 protein levels in asynchronous cultures (C4718) by immunoblotting with an antibody raised against the Cnd1 protein. Immunoblotting against α-tubulin serves as loading control. **(D)** Quantitation of the fraction of mitotic cells that display unequal chromosome segregation at 34°C of strains derived in E; *n* = 258 (*zas1^+^*, *cnd1^+^*), *n* = 231 (*zas1^+^ P_cnd3_-cnd1*), *n* = 101 (*zas1-Δaj3 cnd1^+^*), and *n* = 114 (*zas1-Δaj3 P_cnd3_-cnd1*) mitoses scored. **(E)** Chromosome condensation curves of FROS strains with the indicated genotypes derived by tetrad dissection of one parental diploid strain (C4984). **(F)** Tetrad analysis of a heterozygous *zas1-Δaj3/zas1^+^ P_cnd3_-cnd1/cnd1^+^* diploid fission yeast strain (C4718) imaged after 5 d at 25°C. **(G)** Tetrad analysis of heterozygous *ΔSPBC887.16/SPBC887.16* and *ΔSPBC713.13/SPBC713.13*, *ΔSPBC713.14c/SPBC713.14c* diploid fission yeast strains (C4509, C4508) after 5 d at 25°C. Circles identify the position of spores bearing the *ΔSPBC887.*16 or *ΔSPBC713.13*, *ΔSPBC713.14c* genes, respectively. **(H)** Chromosome condensation curves of FROS strains with the indicated genotypes derived by tetrad dissection of one parental diploid strain (C5048). **(I)** Quantitation of the fraction of mitotic cells that display unequal chromosome segregation at 34°C of strains used in H; *n* = 89 *zas1-Δaj3 P_cnd3_-peg1* mitoses scored; other data from D.

The finding that Zas1 binds to the promoter of the gene that encodes the condensin subunit Cnd1 (Fig. 3 C) and that *cnd1* transcription is reduced to ∼50% in the *zas1-Δaj3* mutant (Fig. 3 D) implied that reduced Cnd1 levels might account for the condensation and segregation defects in *zas1* mutants. As expected, Cnd1 protein levels were substantially reduced in *zas1-Δaj3* cells (Fig. 5 A). The mRNA levels of *cnd2* (Fig. 5 B) and the protein levels of the Cnd2, Cnd3, and Cut3 (Fig. 5 A) condensin subunits were, in contrast, not affected by the *zas1-Δaj3* mutation, which is also consistent with the absence of Zas1-binding peaks and Zas1-UAS motifs in their promoter regions (Fig. S1 D). Furthermore, the assembly of these subunits into condensin complexes was not notably affected in *zas1-Δaj3* mutants (Fig. S1 F). We conclude that Zas1 specifically controls expression of the condensin subunit Cnd1 by activating transcription of the *cnd1* gene.

To test whether reduced Cnd1 protein levels caused the changes in chromosome condensation dynamics in *zas1* mutants, we assayed whether decoupling Cnd1 expression from its regulation by Zas1 would allow normal condensation in *zas1* mutants. Replacement of the *cnd1* promoter with the promoter of the second condensin HEAT-repeat subunit *cnd3* (Fig. S2 B) restored *cnd1* mRNA levels in *zas1-Δaj3* cells to near–wild-type levels (Fig. 5 B). Probing immunoblots of whole-cell extracts with an antibody raised against Cnd1 (Fig. S2 C) showed also that Cnd1 protein levels were equal or even slightly higher in the *zas1* mutants with the promoter replacement than in *zas1^+^* wild-type cells (Fig. 5 C). Whereas promoter replacement did considerably decrease the fraction of *zas1-Δaj3* mutant cells that unequally segregated their chromosomes during anaphase (Fig. 5 D) and completely abolished chromosome arm stretching (Fig. 5 E), it neither rescued the growth defect of the *zas1-Δaj3* mutant (Fig. 5 F) nor accelerated the rate of prophase chromosome condensation in these cells (Fig. 5 E and S2 E). Zas1 must hence have additional essential functions for chromosome condensation timing and cell proliferation that go beyond activating the expression of Cnd1.

### Condensation delays might result from a combinatorial misregulation of Zas1 target genes

Because chromosome condensation and cell proliferation defects in *zas1* mutant cells could not be explained by the misregulation of the *cnd1* gene alone, we systematically assessed the functional importance of other genes that displayed pronounced Zas1-binding peaks in their promoter regions (Fig. 3 B). Genome-wide deletion studies had previously shown that *SPBC1652.02* is not essential for cell proliferation (Kim et al., 2010). We found that deletion of neither the *SPBC887.16* ORF nor the overlapping *SPBC713.13* and *SPBC713.14c* ORFs had any notable effect on cell proliferation (Fig. 5 G). Deletion of any of these genes did also not affect chromosome condensation dynamics (Fig. S2 D). Deletion of *puc1* or any other nonessential genes from the top of the list of Zas1 genomic binding sites, including the ncRNA gene *SPNCRNA.244*; the uncharacterized ORFs *SPAC644.09*, *SPAC18B11.08c*, and *SPAC1565.03*; or the tyrosine phosphatase gene *pyp3*, had no major influence on chromosome condensation dynamics (Fig. S2 D).

We used the same promoter replacement approach that we had applied to uncouple the expression of *cnd1* from Zas1 to other essential genes in the list of highest-ranking Zas1 binding sites, including the TFIIIB complex subunit gene *brf1*, the cell polarity genes *pmo25* and *pob1*, the TF gene *teb1*, the ribosome assembly factor gene *SPAC1B9.03c*, and the 3′-5′-exoribonuclease gene *SPBC609.01*. None of these promoter replacements was able to restore the growth defect of *zas1-Δaj3* mutants (Fig. S2 F). Finally, we tested whether promoter replacement of the *peg1* gene, which encodes a microtubule plus-end–binding protein that is essential for the formation of a stable mitotic spindle and chromosome segregation (Grallert et al., 2006), could restore the chromosome condensation delay or prevent chromosome segregation defects in *zas1-Δaj3* mutant cells. This was, however, not the case (Fig. 5, H and I; and Fig. S2 E).

We conclude that misregulation of a single one of the top Zas1 target genes appears to be insufficient to cause the changes in chromosome condensation dynamics and growth defects in *zas1* mutant cells, which might instead be the consequence of a combined misregulation of several genes controlled by Zas1. Further insights into the mechanism of Zas1 function might thus be required to understand the defects that result from its mutation.

### Zas1 contains a TAD motif that is essential for its function

**Figure 6.**
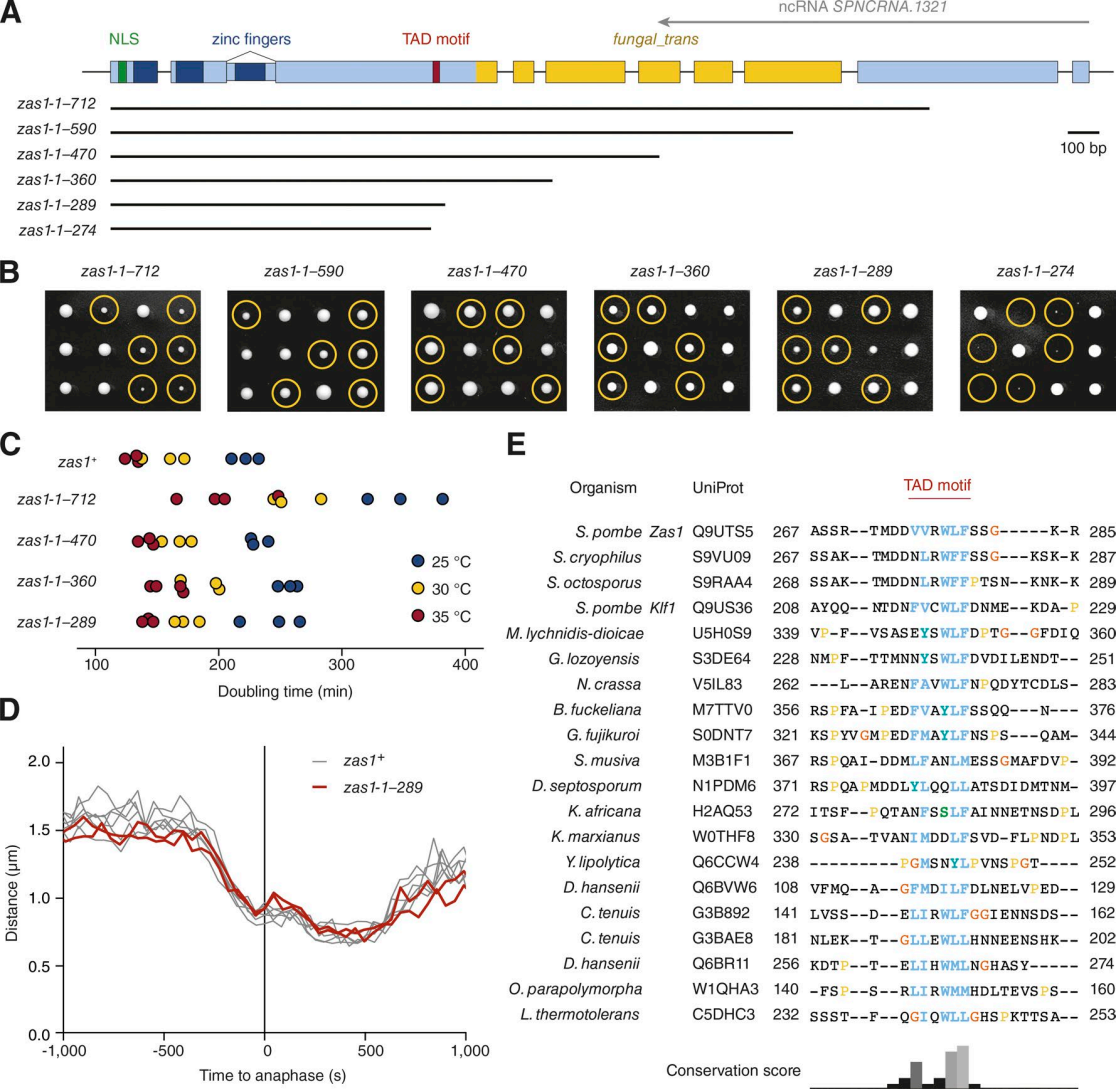
**Identification of a conserved TAD motif in Zas1. (A)** Schematic overview of *zas1* truncation constructs. Numbers indicate the amino acid residue range in each construct. **(B)** Tetrad analysis of heterozygous *zas1-1–x*/*zas1^+^* diploid fission yeast strains (C4006, C4445, C4007, C4036, C4087, C4035) after 5 d at 25°C. Circles identify the position of spores bearing the *zas1-1–x* gene. **(C)** Doubling times of cells expressing truncated versions of the *zas1* gene (C3974, C4026, C4025, C4037, C4047) in rich liquid medium (YE5S) measured in three independent experiments at 25°C, 30°C, and 35°C. **(D)** Condensation curves of cells expressing the truncated *zas1-1–289* (red, C4094, *n* = 2 independent experiments) and wild-type *zas1^+^* control strains (black lines, *n* = 6 independent experiments from Fig. 1). **(E)** Top hits of a protein sequence alignment of Zas1 homologues in different species from a PSI-BLAST search (see supplementary alignment file). Numbers indicate start and end residue numbers of the sequences in the alignment.

To understand how Zas1 controls the expression of its target genes, we turned our attention to the *fungal_trans* domain within the C-terminal half of the protein, which is truncated or mutated in the *zas1* ts mutants (Fig. 2 A). We first tested the functional importance of this protein region by generating an additional set of C-terminal Zas1 truncations (Fig. 6 A). Contrary to our expectation that further shortening of the *fungal_trans* domain would exacerbate the growth defects observed in the *zas1-ts34 (zas1-1–712)* mutant, we found that removal of most of the *fungal_trans* domain (*zas1-1–360*) restored growth to almost wild-type rates (Fig. 6, B and C). This truncation also deleted the complete antisense ncRNA *SPNCRNA.1321* region, which rules out an essential role of this transcript. Cells that expressed a version of Zas1 that lacked the entire *fungal_trans* domain (*zas1-1–289*) similarly grew at near–wild-type rates (Fig. 6 C), segregated chromosomes correctly (Fig. 1 E), and displayed wild-type chromosome condensation kinetics (Fig. 6 D). The *fungal_trans* domain is therefore not strictly required for the essential functions of Zas1.

Further truncation of Zas1 by merely an additional 15 amino acids abolished cell proliferation (*zas1-1–274*; Fig. 6 B). Sequence alignment of Zas1 homologues from different fungal species revealed the presence of a short conserved linear motif with a core of six amino acid residues within the 15-residue region identified in our truncation experiment (Fig. 6 E and supplemental alignment file). The core has a strong hydrophobic and aromatic signature, with a preference for acidic residues to either side. Because proline is disfavored, the motif might be able to fold into an amphipathic two-turn α-helix. Notably, this conserved stretch is reminiscent of short helical amphipathic TAD motifs found in the TFs Gal4, Gcn4, and E2F.

**Figure 7.**
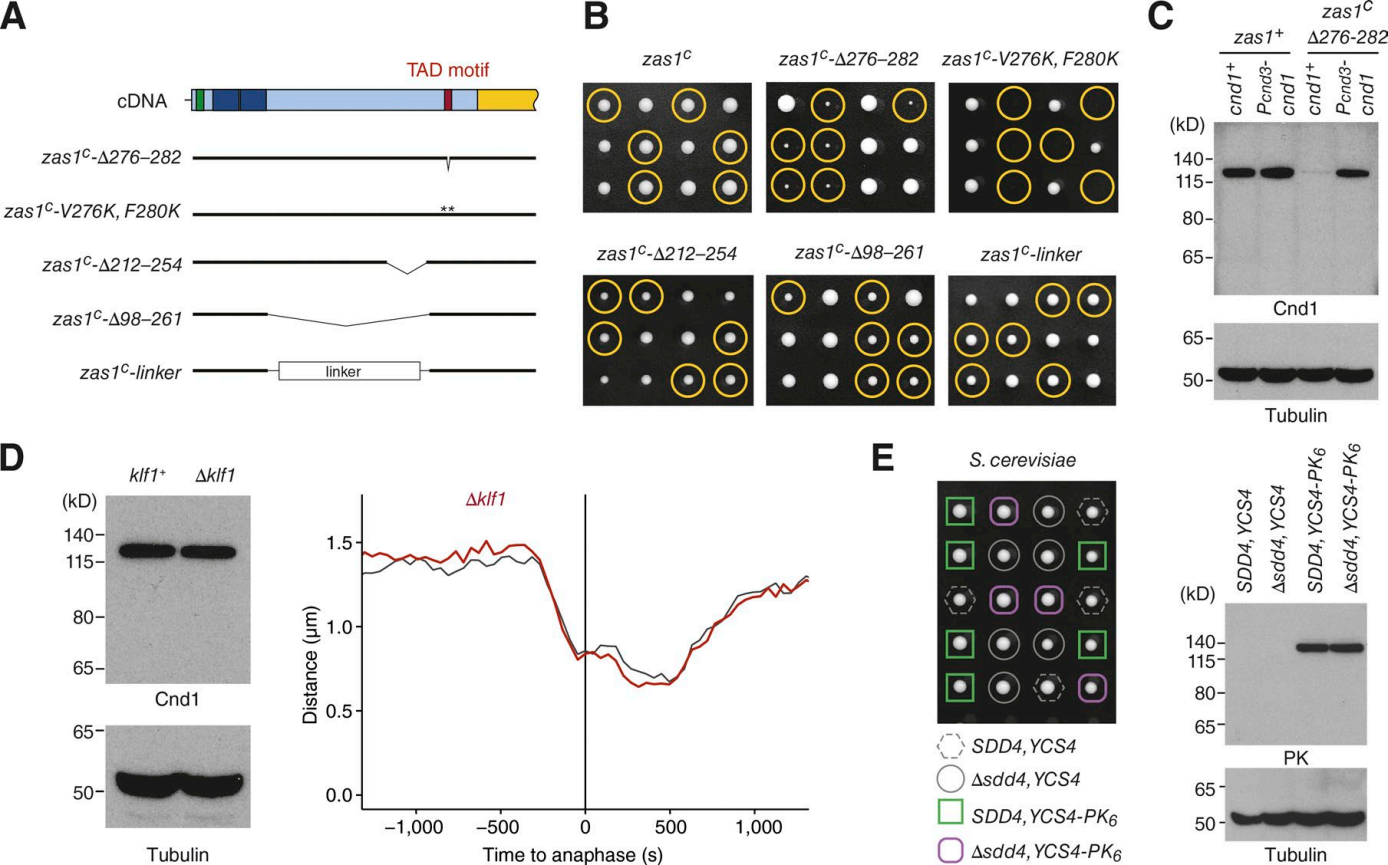
**The TAD motif is essential for the TF function of Zas1. (A)** Overview of mutant versions of *zas1* cDNA alleles. Numbers indicate the amino acid residue range that has been deleted, replaced, or mutated in each construct. **(B)** Tetrad analysis of heterozygous *zas1^c^*/*zas1^+^*, *zas1^c^-Δ276–282*/*zas1^+^*, *zas1^c^-V276K*, *F280K*/*zas1^+^*, *zas1^c^-Δ212–254*/*zas1^+^*, *zas1^c^-Δ98–261*/*zas1^+^*, and *zas1^c^-linker*/*zas1^+^* diploid fission yeast strains (C4083, C4098, C4740, C4387, C4114, C4486) after 5 d at 25°C. Circles identify the position of spores bearing the *zas1^c^*, *zas1^c^-Δ276–282*, *zas1^c^-V276K*, *F280K*, *zas1^c^-Δ212–254*, *zas1^c^-Δ98–261*, and *zas1^c^-linker* genes, respectively. **(C)** Comparison of Cnd1 protein levels in whole-cell extracts of asynchronous wild-type *zas1^+^* and *zas1^c^-Δ276–282* strains expressing Cnd1 from the *cnd1* or from the *cnd3* promoter. Immunoblotting against α-tubulin serves as loading control. **(D)** Comparison of Cnd1 protein levels in asynchronous *klf1^+^* and *Δklf1* cells (C3974, C4573). Immunoblotting against α-tubulin serves as loading control. Comparison of condensation curves of wild-type (black, data from Fig. 5 E) and *Δklf1* (red, C5141, *n* = 161 cells). **(E)** Tetrad analysis of *SDD4/Δsdd4 S. cerevisiae* diploid cells (C4743) after 2 d at 30°C. Comparison of Ycs4-PK_6_ expression levels by immunoblotting against the PK epitope of whole-cell extracts of *SDD4* and *Δsdd4* cells derived from the tetrad dissection.

To test whether the newly identified motif is essential for the function of Zas1, we either deleted the six core consensus residues in the cDNA version of *zas1* (*zas1^c^-Δ276–282*) or mutated two conserved hydrophobic residues to charged side chains (*zas1^c^-V276K*, *F280K*; Fig. 7 A). Cells that expressed either mutant version of Zas1 displayed a strong proliferation defect (Fig. 7 B). Deletion of 42 residues immediately N-terminal of the TAD motif (*zas1^c^-Δ212–254*) had, in contrast, only a minimal effect on cell proliferation. Consistent with the functional importance of the TAD motif for the role of Zas1 in gene regulation, we found that Cnd1 protein levels were drastically reduced in *zas1^c^-Δ276–282* cells, an effect that was again restored by uncoupling transcriptional control of the *cnd1* gene from Zas1 by promoter replacement (Fig. 7 C).

We furthermore assayed for the presence of any additional sequences of functional significance located in the region between the ZFs and the TAD motif. Although deletion of the entire sequence between the two modules resulted in a mild growth defect (*zas1^c^-Δ98–261*; Fig. 7, A and B), this effect might merely be the result of steric hindrance of the TAD motif caused by its spatial proximity to the ZFs. If this were true, we would expect that replacing this region by a random linker sequence would restore proliferation to wild-type levels. This was indeed the case (Fig. 7, A and B). NLS, ZFs, and the TAD motif are hence the only elements required for the essential function of Zas1.

Because the TAD motif is also conserved in the Zas1 paralogue Klf1, we tested whether deletion of *klf1* would affect Cnd1 expression or chromosome condensation. This was, however, not the case (Fig. 7 D), consistent with the notion that Klf1 functions specifically during cellular quiescence (Shimanuki et al., 2013). Furthermore, deletion of the *SDD4* gene, which encodes the closest orthologue of Zas1 found in *S. cerevisiae*, neither impaired cell proliferation nor affected the protein levels of the Cnd1 homologue Ycs4 (Fig. 7 E).

### Identification of a conserved C-terminal helical domain (CHD) in Zas1

**Figure 8.**
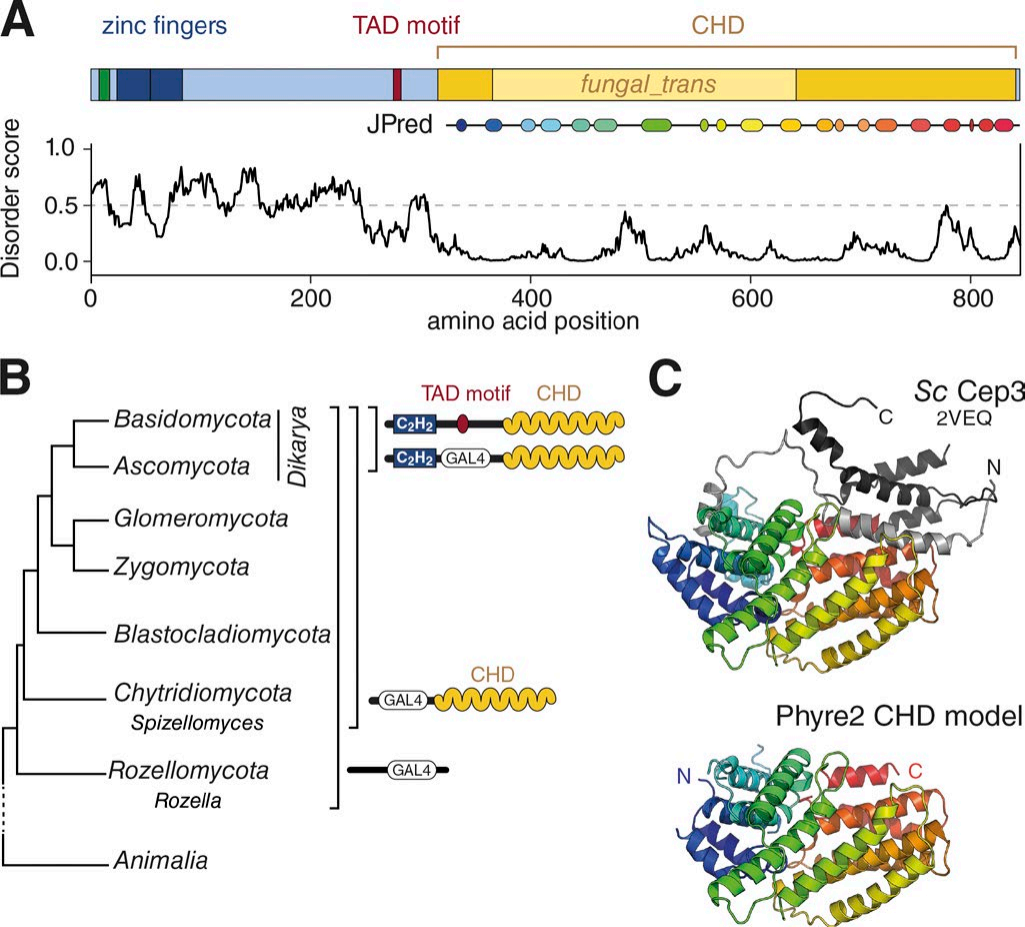
**Zas1 contains a conserved CHD. (A)** Analysis of the Zas1 protein structure. IUPred disorder score and JPred secondary structure prediction of the CHD are indicated. **(B)** Distribution of C_2_H_2_ and Gal4-type (GAL4) ZF proteins containing a CHD during yeast evolution based on a phylogenetic tree after Corsaro et al. (2014). **(C)** Cartoon presentations of the *S. cerevisiae* Cep3 structure (PDB: 2VEQ) and a consensus model for the CHD resulting from a Phyre2 search. N- and C-terminal peripheral helices in Cep3 are shown in light and dark gray, respectively. Images created with Pymol.

These findings raise the question about the role of the large *fungal_trans* domain of Zas1. Secondary structure prediction using the JPred server (Drozdetskiy et al., 2015) suggested that this region of the protein contains a largely α-helical fold that extends to either side of the designated *fungal_trans* domain (Fig. 8 A). We will refer to this domain as the CHD. To gain insights into the function of the CHD, we first attempted to identify proteins with homologous domains. A hidden Markov model (HMM) search based on a sequence alignment of Zas1 homologues was, however, unable to identify proteins outside of the group of *fungal_trans* domain proteins.

When we compared the members of this group, we noticed that most of these proteins contained, in place of the canonical C_2_H_2_ ZFs found in Zas1, fungal-specific Gal4-type Zn_2_C_6_ ZF DNA binding domains (InterPro IPR013087). Whereas in evolutionarily early diverged fungal species, such as *Rozella* spp., Gal4-type ZFs are not linked to any recognizable helical domains, other early diverging fungal clades, such as *Chytridiomycota* (chytrids), as well as all other advanced yeast lineages, possess multiprotein families that contain both Gal4-type DNA binding and helical domains (Fig. 8 B). The genomes of the latter encode proteins that contain both C_2_H_2_ and Gal4-type ZF DNA binding domains, combined with the helical domain. The Gal4-type DNA binding domain was then lost at least once during the evolution of *Dikarya*, the most diverged group of fungi, which resulted in Zas1-like proteins with only C_2_H_2_ ZFs and CHDs (Fig. 8 B).

We took advantage of the finding that Gal4-type ZFs and CHDs are already abundant in primitive chytrids to test whether an alignment of CHDs that represents early events in the creation and amplification of the gene family would be more sensitive in sequence similarity searches than those of the more recently derived Zas1 homologues. An alignment based on a protein from *Spizellomyces* as seed sequence notably improved superposition of predicted α-helices within the CHD (Supplemental Alignment File). Whereas an HMMER search with this new CHD alignment again only retrieved known proteins of the *fungal_trans* group, a search of the HHPred HMM library returned two HMMs as top hits with very strong significance (2VEQ_A and 2QUQ_A). Both HMMs are based on a domain structure of the *S. cerevisiae* kinetochore protein Cep3. The *E* values (1.0 × 10^−33^ and 2.5 × 10^−33^, respectively) are remarkably low, taking into account that the sequence identity between *S. cerevisiae* Cep3 and *S. pombe* Zas1 is in the random similarity zone (11% identity, optimized by gap insertion).

In a second approach, we used the *Spizellomyces* seed sequence in the Phyre2 threading/HMM tool (Kelley et al., 2015) and retrieved crystal structures of the Cep3 CHD with good confidence (Bellizzi et al., 2007; Purvis and Singleton, 2008). Phyre2 furthermore provided a structural model for the *Spizellomyces*-based sequence alignment (Fig. 8 C). In comparison to the CHD model, Cep3 contains peripheral N- and C-terminal helices whose absence in the CHD model does, however, not disrupt the main fold. We conclude that the CHD fold represents the core ancestral domain. Further query of the Dali structure similarity server (Holm and Laakso, 2016) with the Cep3 structure identified no additional structural relatives.

### The Zas1 TAD motif binds the CHD

**Figure 9.**
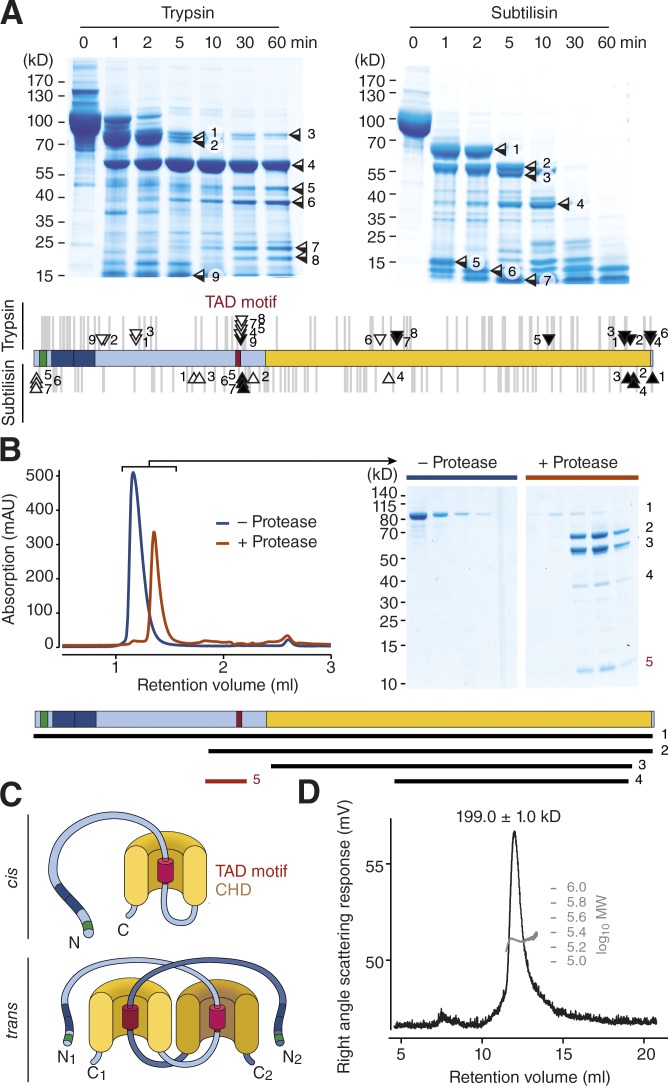
**The Zas1 TAD motif binds to the CHD. (A)** Coomassie-stained SDS PAGE of Zas1 proteolysis products of trypsin or subtilisin digestion for the indicated time periods. The identity of the main cleavage products was determined by mapping N (open boxes/arrows) and C (closed boxes/arrows) termini using mass spectrometry. Gray lines indicate the positions of theoretical trypsin (top) or subtilisin (bottom) cleavage sites in the protein. **(B)** Analytical SEC before (blue curve) or after (red curve) limited proteolysis of Zas1 with trypsin. Peak fractions (gray bars) were analyzed by SDS-PAGE and Coomassie staining. The identities of the bands were determined by mapping N and C termini using mass spectrometry and are indicated in the scheme. **(C)** Models of the interaction of the TAD motif (red) with a helical domain (yellow) of the same Zas1 molecule (in cis) or with a second Zas1 molecule (in trans). **(D)** SEC-MALS analysis of purified Zas1 (mean molecular weight estimate ± min/max from two independent chromatography runs).

Short linear motifs located in disordered regions frequently bind to globular domains of other proteins in trans to create regulatory networks or of the same polypeptide chain in cis to control protein activity by conformational changes, a mechanism that is frequently used by kinases (Gibson and Kumar, 2016). We speculated that the TAD motif in Zas1 might function as such a regulatory motif. One important prediction of this hypothesis is that the motif needed to be surface-exposed to interact with globular domains. To test this prediction, we performed limited proteolysis of purified Zas1 with two different proteases and identified the boundaries of the cleavage fragments by mass spectrometry (Fig. 9 A). Consistent with the notion that the TAD motif is accessible to other proteins, a large portion of the fragment boundaries mapped within the vicinity of the TAD motif.

We then tested whether the Zas1 TAD motif binds to the Zas1 CHD. We again subjected purified Zas1 to limited proteolysis, but this time stopped the reaction after 2 min. Cleavage under these conditions produced fragments that contained the TAD motif and the CHD, only the CHD, or only the TAD motif (Fig. 9 B, fragments 2, 3, or 5). Remarkably, the small fragment that only contained the TAD motif (fragment 5) coeluted with the considerably larger fragments that contained the CHD (fragments 2 and 3) in size exclusion chromatography (SEC), which can be explained only if the TAD motif fragment bound to the fragments of Zas1 that contained the CHD.

The TAD motif of Zas1 might either bind in cis to the CHD of the same molecule or in trans to the CHD of a second Zas1 molecule (Fig. 9 C). To test for the possibility of trans-binding, we obtained an accurate estimate of the molecular mass of the purified Zas1 protein by analytical SEC coupled to multiangle light scattering (SEC-MALS; Fig. 9 D). The mass estimate of 199 kD very well matched the predicted molecular weight of a Zas1 homodimer (2 × 96.8 kD). Unfortunately, we were not able to obtain sufficient amounts of Zas1 protein with a deletion of the TAD motif to directly test the role of the motif in dimer formation, presumably because the mutant protein is unstable. We conclude that the TAD motif and the CHD of Zas1 can interact, either in cis to create an intramolecular conformational switch or in trans to mediate the interaction between two (or potentially more) Zas1 proteins. Because, however, the CHD is dispensable for cell proliferation (Fig. 6, A and B), whereas the TAD motif is not (Fig. 7, A and B), it is very likely that the TAD motif also mediates the interaction with another yet-unidentified Zas1 partner protein.

## Discussion

### The Zas1 TF controls chromosome condensation dynamics

In this study, we identified the ZF TF Zas1 as a key regulator of mitotic chromosome condensation dynamics in fission yeast and demonstrated that it controls the expression of a specific set of genes, including the *cnd1* gene that encodes one of the two HEAT-repeat subunits of the condensin complex. A recent study found that, in budding yeast, expression of the other HEAT-repeat subunit is down-regulated during G1 phase of the cell cycle, which limits condensin binding to chromosomes (Doughty et al., 2016). Might Zas1 control the expression of Cnd1 in a similar cell cycle–dependent manner in fission yeast? Although *cnd1* mRNA levels have been reported to increase during late G2 and M phases (Rustici et al., 2004; Peng et al., 2005), no extensive changes in the abundance of the Cnd1 protein (or any other condensin subunit) have been found in a quantitative proteome-wide analysis of fission yeast cells at different stages of the cell cycle (Carpy et al., 2014). Even if Zas1 activated *cnd1* transcription only in preparation for mitosis, such a cell cycle–dependent control would probably not be essential for condensin function, because replacement of the *cnd1* promoter with the *cnd3* promoter, which shows no cell cycle–dependent regulation (Rustici et al., 2004; Peng et al., 2005), has no effect on chromosome condensation or segregation (Fig. 5, D and E). Since the nuclear localization of condensin in *S. pombe*, in contrast to *S. cerevisiae*, is restricted to mitosis (Sutani et al., 1999), a cell cycle–dependent transcriptional regulation of one its subunits might simply be unnecessary in this species.

Despite the fact that uncoupling transcription of *cnd1* from Zas1 control restored *cnd1* mRNA and Cnd1 protein concentrations in *zas1* mutant cells to wild-type levels, it did not fully rescue the defects in either chromosome condensation dynamics or chromosome segregation (Fig. 5, D and E; and Fig. S2 E). Uncoupling the expression of another set of essential genes regulated by Zas1 similarly failed to restore normal proliferation of *zas1* mutants (Fig. 5, H and I; and Fig. S2, E and F), and deletion of nonessential Zas1 target genes did notproduce condensation or segregation defects (Fig. S2 D). The lack of success in assigning these defects to a single gene suggests that Zas1 either controls the expression of yet another target gene with an essential role that is still to be identified or, more likely, that the condensation delay and chromosome segregation phenotypes are the accumulative result of a simultaneous misregulation of multiple target genes.

### Implications for the mechanism of chromosome condensation and decondensation

Albeit with slower dynamics, chromosomes reach the same level of compaction at metaphase in *zas1* mutant cells as they do in wild-type cells (Fig. 1 D), despite markedly decreased Cnd1 expression levels (Fig. 5 A). The finding that a reduction in the number of functional condensin holocomplexes has no influence on the final compaction state is consistent with the notion of a catalytic, rather than a mere structural function of the complex. If condensin complexes organize chromosomes by extruding DNA loops, as recent chromosome conformation capture (Naumova et al., 2013; Gibcus et al., 2018) and single-molecule imaging experiments (Terakawa et al., 2017; Ganji et al., 2018) imply, then it might be conceivable that a smaller number of condensin complexes could simply create larger-sized loops to achieve the same degree of compaction.

Nevertheless, chromosomes assembled in the presence of a lower number of condensin complexes frequently fail to partition correctly during the ensuing anaphase (Fig. 1, E and F). It is hence conceivable that the number of linkages that can be generated by fewer condensin holocomplexes becomes limiting only when they need to provide chromosome arms with the mechanical stability required for their segregation (Hirota et al., 2004; Gerlich et al., 2006; Oliveira et al., 2007; Cuylen et al., 2013). Alternatively, changes in the dynamics of chromosome condensation might prevent complete decatenation of sister chromatids by topo II (Piskadlo et al., 2017). Both possibilities are consistent with the finding that restoring Cnd1 levels by promoter replacement reduces the number of missegregation events (Fig. 5 D).

Interestingly, in *zas1* mutant cells, the rate of chromosome decondensation upon exit from mitosis decreases to a similar degree as the condensation rate decreases during mitotic entry (Fig. 1 F). If decondensation were initiated by the release of condensin-mediated DNA linkages, we would expect to observe faster rather than slower decondensation kinetics in cells with lower levels of intact condensin holocomplexes. It therefore seems likely that chromosome decompaction upon exit from mitosis is an active process that requires more than the mere inactivation of condensin. This notion is consistent with the conclusions from studies of chromosome decondensation in *Xenopus* egg extracts (Magalska et al., 2014).

### The molecular mechanism of transcriptional control by Zas1

The genes controlled by Zas1 seem to be involved in surprisingly divergent cellular pathways. What Zas1 target genes have in common, however, is the presence of one or multiple copies of a 6-bp consensus UAS in the vicinity of their transcription start sites, which is presumably bound cooperatively by both Zas1 ZFs (Pavletich and Pabo, 1991). Because the promoters of Zas1 target genes frequently contain two or more Zas1-UAS motifs (Fig. 4 B), it is conceivable that multiple copies of Zas1 bind to these promoters simultaneously, possibly as dimers, as our in vitro experiments suggest (Fig. 9 D). A second commonality of Zas1-binding sites is their localization to the arms of chromosomes I and II, with an apparent depletion in regions of ∼0.8 Mb around centromeres (Fig. 3 A). Although we found only one of the top 60 Zas1-binding sites located on chromosome III, it remains to be determined whether Zas1 could function as a chromosome-specific TF.

How might Zas1 control the expression of its target genes? We identified a conserved motif in the Zas1 amino acid sequence that resembles the consensus sequences found within the TADs of several TFs, including the metazoan cell cycle regulator E2F and yeast Gal4 and Gcn4 (Fig. 6 E; Piskacek et al., 2016; Staby et al., 2017). The presence of a functionally important TAD motif suggests that, as in most other known fungal transcriptional activators, this motif might bind to the KIX domain of the Gal11/Med15 subunit of the mediator complex. Despite extensive efforts, we were unable to identify Zas1 interaction partners using a range of biochemical approaches (unpublished data). It is conceivable that interactions with the Zas1 TAD motif might take place only when the protein is bound to chromosomes, and/or the interactions might be very transient and therefore escaped our copurification attempts. The large size of the 21-subunit mediator complex and its association with chromatin might furthermore explain the failure to recover mediator in Zas1 pull-down experiments.

Regardless of the fact that binding of TAD motifs to KIX domains appears to represent a general transcriptional activation system, proteins bearing the KIX domains can have very different architectures (Piskacek et al., 2016; Staby et al., 2017). Moreover, the short amphipathic helices formed by all TAD motifs can bind to the structurally flexible KIX domains in different orientations. Because of this variability, despite fulfilling all the sequence requirements of a KIX-binding TAD motif, future experiments will have to address whether Zas1 functions as a bona fide TAD protein that can activate transcription at its binding sites in the fission yeast genome by recruiting other protein partners.

### A conserved TAD–CHD module as a cis/trans switch mechanism?

Although we were unable to identify other binding partners of the Zas1 TAD motif, we discovered that it binds to the large α-helical domain in the C-terminal half of Zas1 (Fig. 9 B). We predict that the mutations in the CHD identified by us and others destabilize this domain fold, which would explain their ts phenotype. Because of the similarity of the Zas1 TAD motif to the TAD motif of E2F, we initially favored the idea that the CHD might be homologous to the cyclin fold found in the retinoblastoma protein Rb to which the E2F TAD motif binds (Lee et al., 2002). Our HMM searches revealed, however, that the fold of the CHD instead resembles the helical domain found in TFs of the Gal4 superfamily, which is represented by the structure of the *S. cerevisiae* kinetochore subunit Cep3 (Fig. 8 C). Because Cep3 is present only in *Saccharomyces* and closely related species, it is most likely a highly derived and late-evolved member of the family of proteins containing a Gal4-type DNA binding motif and a CHD, from which Zas1 eventually emerged by replacement of the Gal4-type with canonical C_2_H_2_ ZFdomains (Fig. 8 B).

What might be the function of the TAD–CHD interaction? One possibility is that binding of the TAD motif to the CHD in cis shields the motif from interacting with its trans-activating binding partners, such as mediator, in a manner similar to how kinases are held inactive by an autoinhibitory conformation (Gibson and Kumar, 2016). If this were true, it is conceivable that the switch from a cis to a trans interaction could be triggered by posttranslational modifications, for example phosphorylation. Alternatively, binding of the TAD motif of one Zas1 molecule to the CHD of a second Zas1 molecule in trans might serve the formation of Zas1 homodimers (Fig. 9 C) or, in quiescent cells, Zas1–Klf1 heterodimers (Shimanuki et al., 2013). However, because neither the Zas1 CHD nor Klf1 is essential for cell viability or efficient chromosome condensation (Figs. 6 B and 7 D), we favor the hypothesis that the Zas1 TAD motif interacts with yet-unknown partner proteins. Identification of these binding partners and insights into the regulatory principles that control Zas1 activity will be essential future tasks for revealing the mechanisms that guide the control of mitotic chromosome condensation and for gaining insights into the evolutionarily conserved aspects of TF function.

## Materials and methods

### Yeast strain construction

Point mutations were introduced into the *zas1* gene using a loop-in/loop-out strategy. The *zas1* ORF was cloned into pUR19, which contains the *S. pombe ura4^+^* marker gene cassette. Point mutations were introduced into this plasmid using site-directed mutagenesis (QuickChange; Agilent Genomics). The plasmid was then linearized and introduced into yeast strain C1283 using a LiAc-PEG/DMSO transformation protocol (Murray et al., 2016a). After negative selection on 5-FOA plates to select for clones in which both *zas1* copies had recombined, the presence of the mutations was validated by Sanger sequencing. The *zas1* mutant strains were crossed to a strain containing the FROS (C2926; Petrova et al., 2013), sporulated, and dissected.

C-terminal fluorescent protein or epitope tags were fused to the endogenous *zas1*, *cnd1*, *cnd2*, or *cnd3* ORFs by PCR targeting as described previously (Bähler et al., 1998), using pFA6a PK_6_-kanMX6 (for PK_6_-tagging), pFA6a GFP-kanMX6 (for GFP-tagging; Longtine et al., 1998), or pFA6a zas1-tdTomato-kanMX6 and primers listed in Table S2.

Truncations of the *zas1* gene were created by PCR amplification of a kanMX4 resistance cassette (Wach et al., 1994) with the homology primer pairs listed in Table S2 to introduce a stop codon at the desired position of the *zas1* ORF. After integration at one *zas1* allele in a diploid strain and verification of correct integration by colony PCR and Sanger sequencing, cells were grown on rich medium overnight and patched onto SPAS medium to induce sporulation for 24 h at 25°C. Asci were transferred to YE5S plates and incubated for 16–24 h at 25°C. Tetrads were dissected using a Singer MSM microscope, and plates were incubated for 3 d at 30°C or 5 d at 25°C (for ts mutants).

A *zas1^c^* cDNA allele was created by reverse transcription of an *S. pombe* mRNA pool using Superscript III (Invitrogen) according to the manufacturer’s instructions, amplification by PCR, and integration into a pUC plasmid backbone. The *zas1* promoter (385 bp 5′ of the *zas1* start codon) and the *zas1^+^* terminator (300 bp 3′ of the *zas1* stop codon) were added, and a natMX6 resistance cassette was inserted 3′ of the terminator sequence, resulting in pNat zas1(cDNA). The zas1(cDNA)-natMX6 gene was amplified via PCR (primers zas1for/zas1rev; Table S2) and introduced into the *zas1* locus by homologous recombination.

To replace promoters with the promoter of *cnd3*, the 392 bp 5′ of the *cnd3* start codon were inserted 3′ of the kanMX cassette in pFA6a kanMX4 to create pFA6a kanMX4-Pcnd3. The kanMX4-Pcnd3 construct was PCR-amplified with primers listed in Table S2 and transformed into *S. pombe* strain C2457 (Table S1).

### Yeast strain validation

Colonies appearing after transformation were screened for correct integration by colony PCR using a forward primer binding 5′ outside of the integration site and a reverse primer inside the integration site. Cells from positive colonies were singled out by streaking on the appropriate selective agar plates. Colonies formed from single cells were tested for correct integration by a second colony PCR using a forward primer binding inside the integration site and a reverse primer binding 3′ of the integration site. Genomic DNA was isolated from haploid strains grown in liquid medium (Murray et al., 2016b), and the integration site was PCR-amplified with Phusion proofreading polymerase (Thermo Fisher) using primers binding 5′ and 3′ outside the integration site. The PCR product was Sanger sequenced.

To isolate strains after transformations of diploid cells, up to 10 clones tested by colony PCR for integration were grown on the respective medium at 34°C and tetrad dissected. Colonies originating from tetrad dissection were replica plated onto appropriate selective medium and genotyped after 3 d at 25°C. To confirm correct integration, genomic DNA was extracted and used as a template for two PCR reactions with (a) a forward primer 3′ of the integration site and a reverse primer inside the integration site and (b) a forward primer binding inside the integration site and a reverse primer binding 3′ of integration site. Both PCR reactions were Sanger sequenced.

### Live-cell imaging

Strains expressing the respective FROS labels (Petrova et al., 2013) were prepared for imaging by lactose gradient enrichment of G2 cells and creation of a cell monolayer as described (Schiklenk et al., 2017). Cells were imaged in YE5S medium on an Olympus Cell^R^ microscope system based on an IX83 frame equipped with 488- and 561-nm lasers (Coherent), quadruple band dichroic filters (FWHM 440/60, 525/50, 600/40, 655LP; Semrock), and a Hamamatsu C9100-13 EM-CCD camera and through an Olympus UApo N 100× OTIRF (NA 1.49) oil objective. Chromatic aberrations were confirmed to be insignificant for the Apochromat objective. 10 z-positions with 400-nm focal-plane spacing were acquired for up to six different positions every 45 s. For each focal plane, images of the green and red channel were acquired before proceeding to the next z-position. The z-focus was stably maintained by an Olympus ZDC-3 z-drift compensator. Camera, lasers, shutters, and stage were controlled by CellSens acquisition software. Cells were imaged at 34°C, except those in Video 1 and Fig. 2 C, which were acquired at 25°C.

To coimage Hht2-GFP and Zas1-tdTomato, optical sections (0.9-µm thickness) of a monolayer of *S. pombe* cells were recorded on a confocal Zeiss LSM 880 microscope using 488- and 561-nm excitation lasers, respectively. Nucleolar and nonnucleolar regions were manually segmented based on the green channel. Segmentations were used to measure mean pixel intensity in the red channel.

### Image analysis

FROS foci positions were extracted using a custom ImageJ plugin (Petrova et al., 2013). Segmentation accuracy and time points of anaphase onset were manually determined for each cell by visual inspection. Distance values from incorrectly segmented frames were discarded. Distance–time series were analyzed in R (R Core Team) using the dplyr package (Wickham et al., 2018) by averaging distance values for each time point in respect to anaphase onset. Averages and standard deviation were plotted using the ggplot2 package (Wickham, 2009).

The *nls* function of R (Bates and Chambers, 1992) was used to fit the sigmoid function

$$d=\frac{k}{1+e^{at+b}}+d_{offset}$$

to each condensation curve (time points –1,800 to 0 s) as previously described (Petrova et al., 2013), except that the contribution of each data point to the fit was weighted by the number of measurements of each time point.

### RT–quantitative PCR (qPCR)

Cells of the desired genotype from three independent tetrads were cultured in YE5S at 25°C to OD_600_ 0.8–1.0, and total RNA was isolated from 7.5 × 10^7^ cells by phenol/chloroform extraction. Two RT reactions per sample were performed with 1 µg RNA per reaction using Maxima First Strand cDNA Synthesis Kit (Thermo Fisher). Transcript abundance was measured by qPCR using SYBR Green Mix (Thermo Fisher) on a StepOnePlus thermocycler (Applied Biosystems) using primer pairs described in Table S2, and for *act1* as described previously (Helmlinger et al., 2008). For each qPCR run, primer efficiency was determined by RT reaction serial dilution. CT values were calculated with StepOne software (Applied Biosystems). Relative cDNA abundance was calculated as described previously (Pfaffl, 2001).

### ChIP

ChIP was performed as described (Sutani et al., 2015). Cells were grown at 30°C in 200 ml YE5S to OD_600_ 1.0. 22 ml freshly prepared formaldehyde fixation solution (25 mM Tris-HCl, pH 8.0, 100 mM NaCl, 1 mM EDTA, 0.5 mM EGTA, and 12% formaldehyde) was added, and the culture was incubated for 10 min at 26°C in a shaking water bath. Then, 22 ml of 2.5 M glycine solution was added, and the mixture was chilled on ice for 30 min with occasional shaking. The cell suspension was pelleted at 1,000 *g* and 4°C for 3 min, resuspended in ChIP buffer 1 (Hepes-KOH, pH 7.5, 140 mM NaCl, 1 mM EDTA, 1% Triton X-100, and 0.1% sodium deoxycholate), and washed once with ChIP buffer 1 and once with ChIP buffer 1 containing protease inhibitors (2× cOmplete and 1 mM PMSF) before flash-freezing the pellet in liquid nitrogen.

After thawing, cells were resuspended in 250 µl ChIP buffer 1 containing protease inhibitors and broken by glass bead lysis during five cycles (60 s at 6.5 m/s, 3-min cooling on ice between each cycle; MP FastPrep). Lysis efficiency was confirmed by microscopy, and 130 µl suspension was transferred to a microTUBE AFA (520045; Covaris). Chromatin was sheared by sonication (S220; Covaris) at intensity 4, duty factor 10%, and 200 cycles per burst for 100 s at 4°C. The sonicated sample was cleared by 15-min centrifugation at 14,000 *g* and 4°C, and the supernatant was transferred to a fresh tube. Protein concentration was measured by Bradford assay. All samples were diluted to the lowest sample concentration (maximum 20 mg/ml) with ChIP buffer 1. As input samples, 25 µl samples were set aside and stored at –20°C. To 600 µl of the remaining sample, 1 µg V5 antibody (MCA1360; Bio-Rad) was added, followed by 1-h incubation at 4°C, addition of 10 µl Dynabeads Protein G (10003D; Thermo Fisher), and an additional 1-h incubation at 6°C on a rotating wheel. Beads were collected using a magnet, washed once with 200 µl and twice with 1 ml ice-cold ChIP buffer 2 (50 mM Hepes-KOH, pH 7.5, 500 mM NaCl, 1 mM EDTA, 1% Triton X-100, and 0.1% sodium deoxycholate), then once with 200 µl and twice with 1 ml ice-cold ChIP buffer 3 (50 mM Tris-HCl, pH 8.0, 250 mM LiCl, 1 mM EDTA, 0.5% NP-40, and 0.5% sodium deoxycholate). Finally, beads were washed with 1 ml ice-cold TE buffer (50 mM Tris-HCl, pH 8.0, and 10 mM EDTA) and transferred to a fresh tube. Dynabeads were pelleted and resuspended in 75 µl TES buffer (50 mM Tris-HCl, pH 8.0, 10 mM EDTA, and 1% SDS).

The volumes of the input (25 µl) and immunoprecipitation (75 µl) samples were adjusted to 110 µl with TES buffer. To each sample, 3 µl of 10 mg/ml RNaseA solution (Roche) was added, followed by 15-min incubation at 37°C, and then 3 µl of 20 mg/ml Proteinase K solution was added, followed by an additional 30-min incubation at 37°C. SDS was adjusted to 2.5%, and samples were incubated at 65°C overnight. A one-tenth sample volume of 3 M sodium acetate solution, pH 7.0. was added before addition of one sample volume of phenol/chloroform/isoamyl alcohol (Roth). Samples were vortexed for 30 s and incubated for 10 min at 65°C, followed by centrifugation for 5 min at 21,000 *g*. The aqueous phase was transferred to a fresh tube, and mussel glycogen (10901393001; Roche) was added to a final concentration of 0.125 mg/ml. Two sample volumes of ethanol absolute were added, and DNA was precipitated at –20°C for at least 30 min. DNA precipitates were pelleted by centrifugation for 15 min at 21,000 *g* and 4°C, washed with 1 ml of 70% ethanol, and air-dried at room temperature. DNA pellets were resuspended in ddH_2_O, and DNA concentrations were determined using a Qubit fluorimeter. DNA yield was in the range of 1–10 ng per sample.

### NGS library preparation, sequencing, and ChIP-seq analysis

Library preparation for two independent biological replicates per sample was performed using the NEBNext ChIP-Seq Library preparation kit (E6240L; NEB) according to the manufacturer’s instructions. DNA purification was performed after each reaction using SPRI beads (B23318; Coulter Beckman). The library was indexed and amplified with NEBNext Multiplex Oligos Set 1 (E7335L; NEB) in 11 cycles of a PCR reaction. The amplification product was enriched for 200-bp fragments by gel elution using the e-gel system (G6500 and G6512; Thermo Fisher). The library was single-end sequenced on an Illumina HiSeq2500 for 50 cycles.

Sequencing data were analyzed on the European Molecular Biology Laboratory (EMBL) *Galaxy* platform (Afgan et al., 2016) using a generic ChIP-seq analysis workflow. In short, reads were mapped to *S. pombe* genome assembly (McDowall et al., 2015) using *Bowtie2* (Langmead and Salzberg, 2012). Non-aligning reads were discarded, and duplicate reads were kept. Aligned reads were used to create *bigwig* alignment maps. ChIP peaks were determined using *MACS 1.4* (Feng et al., 2011) with the following parameters: genome size, 12.57 × 10^6^ bp; single-end mode; tag size, 50 bp; bandwidth, 110 bp; M-fold, 10–30; and peak detection P value cutoff 10^−5^, using the input sample for calculation of λ_local_. Lists of peaks regions from both replicates were compared using the *Galaxy* implementation of IDR (Landt et al., 2012). Peaks that were also present in untagged samples were deleted manually to obtain the final list of binding sites.

### Gene ontology term analysis

The list of genes with promoter regions enriched more than 6.0 times in the Zas1 ChIP-seq data (*SPBC887.16*, *SPBC1652.02*, *SPBC713.14C*, *SPBC713.13*, *SPBC19F5.01c*, *SPNCRNA.217*, *SPAC3G9.12*, *SPNCRNA.244*, *SPAC644.09*, *SPAC18B11.08c*, *SPAC17H9.16*, *SPBC13E7.10c*, *SPBC13E7.11*, *SPAC1834.06c*, *SPAC11E3.09*, *SPBC776.13*, *SPAC1565.03*, *SPACB9.03c*, *SPAC13G7.10*, *SPBC1289.04c*, *SPAC6G10.04c*, *SPAC3C7.14c*, *SPNCRNA.337*, *SPBC23G7.08c*, *SPAC56F8.15*, *SPNCRNA.1375*, *SPAC56F8.13*, *SPNCRNA.253*, *SPNCRNA.86*, *SPAC4G8.11c*, and *SPNCRNA.1070*) was analyzed using AnGeLi (Bitton et al., 2015) using the following parameters: predefined background, protein-coding genes; gene ontology category, biological process; adjust method for multiple testing, none; show results with P value smaller than 0.01; and perform pairwise interactions enrichment analysis, no.

### Identification of Zas1-UAS

Sequences of the Zas1 ChIP-seq peaks (Table S2) were submitted to both MEME (v.4.12.0; Bailey and Elkan, 1994) and DMINDA2 (Yang et al., 2017) servers. DMINDA2’s motif-finding algorithm was used with standard parameters; MEME was used with the following parameters: normal mode; alphabet, DNA; site distribution, zero or one occurrence per sequence; number of motifs to be found, 3; minimum motif width, 4; and maximum motif width, 20. To identify Zas1-UAS in ChIP peaks, the *S. pombe* genome (PomBase) was forged into a BSgenome package as described in the package documentation (Pagès, 2017). The genome DNA sequences from the intervals of the ChIP peaks (Table S1) was retrieved, and the instances of the motif were counted using the *stringr::str_count()* function.

To predict DNA binding sites in the Zas1 ChIP-seq peaks (Table S2) de novo, these sequences together with the Zas1 amino acid sequence (Uniprot Q9UTS5) were submitted to the NA binding site predictor for C_2_H_2_ ZF proteins (zf.princeton.edu).

### Condensin complex purification

Strain C5183 was tetrad dissected, and spores bearing the *cnd2-PK_6_* allele and *zas1^+^* or *zas1-Δaj3*, *P_cnd3_-cnd1* or *cnd1^+^* were identified by colony PCR. Colonies with desired genotypes were patched onto YE5S and grown at 25°C. Patches were used to inoculate 50-ml YE5S starting cultures of each strain. The cultures were diluted to OD_600_ 0.1 in 1 liter YE5S medium and grown to OD 1 at 25°C. Cells were harvested by centrifugation, washed three times with 50 ml ice-cold PBS, and resuspended in 7 ml lysis buffer (150 mM Tris-HCl, pH 8.0, 150 mM NaCl, 0.1% NP-40, 1 mM DTT, 1 mM PMSF, and 1× cOmplete protease inhibitor; Roche). Drops of the suspension were flash-frozen in liquid N_2_. Cells were lysed in a cryomill (SPEX) for five 3-min cycles with 10 cps and 2-min cooling after each cycle. Lysate powder was thawed at 4°C by addition of 10 ml lysis buffer supplemented with 14 µg/ml DNase I (Roche). Lysates were cleared by centrifugation for 20 min at 20,000 *g* at 4°C. 4 µg mouse anti-V5 antibody (AbD Serotec) was added to each sample and incubated for 1 h at 4°C on a rotating wheel. 25 µl DynaBeads protein A (Thermo Fisher) was added, and the samples were incubated for an additional hour at 4°C on a rotating wheel. Beads were collected using a magnet and washed four times with 1 ml lysis buffer. Samples were eluted in 12 µl of 5× Laemmli buffer (1 M Tris-HCl, pH 6.8, 5% SDS, 50% glycerol, 0.05% bromophenol blue, and 1 mM DTT) and analyzed using SDS-PAGE (4–12% Bis-Tris gradient gel [Thermo Fisher] in MOPS running buffer). Proteins were visualized using silver staining.

### Zas1 overexpression and purification

The short *zas1* cDNA ORF was cloned into pFastBac His_6_-TEV. This construct was used to create a baculovirus and overexpress Zas1 in Sf9 insect cells as described (Piazza et al., 2014). The cell pellet from 100 ml Sf9 cell culture was resuspended in 30 ml lysis buffer (50 mM Tris-HCl, pH 7.5, 500 mM NaCl, 5 mM β-mercaptoethanol, 25 mM imidazole, 1× cOmplete protease inhibitor [EDTA-free; Roche], and 1 µM PMSF) and sonicated (3× 45 s with 30-s pauses, Branson Sonifier 250, duty cycle 50%, output 5) on ice. The lysate was cleared by 20-min centrifugation at 20,000 *g* and 4°C and incubated with 3 ml Ni-Sepharose 6FF (GE Healthcare) for 1 h at 6°C. Beads were pelleted by 2-min centrifugation at 1,200 *g* and 4°C and washed three times with 15 ml lysis buffer. Bound protein was eluted in 3-ml fractions of elution buffer (50 mM Tris-HCl, pH 7.5, 500 mM NaCl, 5 mM β-mercaptoethanol, 250 mM imidazole, 1× cOmplete protease inhibitor, and 1 µM PMSF). Elution fractions were pooled and dialyzed overnight at 4°C against 2 liter dialysis buffer (100 mM Tris-HCl, pH 8.3, at 4°C, 375 mM NaCl, and 2 mM DTT). Protein samples were concentrated using a Vivaspin 20 column (30 kD cutoff; Satorius) and further purified using SEC column Superdex 200 Increase 10/300 GL on an Äkta Purifier system. SEC fractions containing Zas1 as judged by SDS-PAGE and Coomassie staining were pooled and concentrated using a Vivaspin 20 concentrator (30 kD cutoff; Satorius).

### Electrophoretic mobility shift

5′ 6-carboxy-fluorescein–labeled oligonucleotides (Merck; Table S2) were annealed with unlabeled reverse complement oligonucleotides at a final concentration of 20 µM in a thermocycler using a temperature gradient of 0.1°C/s from 95°C to 4°C. The annealed oligonucleotides were diluted to a final concentration of 400 nM in binding buffer (10 mM Tris-HCl, pH 8.0, 125 mM NaCl, 5 mM MgCl_2_, and 20 µM ZnCl_2_) before the addition of recombinant Zas1 protein to final concentrations of 0, 200, 400, and 800 nM. The mixture was incubated for 90 min on ice in the dark before addition of glycerol to a final concentration of 4.3% (vol/vol) and loaded onto a 1.8% (wt/vol) agarose gel. Electrophoresis was performed in Tris-acetate buffer (pH 8.7 at 6°C) for 14 h at 55 V (0.56 V/cm) at 6°C in the dark. The gel was analyzed on a Typhoon FLA 9500 scanner (GE Healthcare) with excitation at 473 nm with LPB (510LP) filter settings.

### Limited proteolysis

1 µl of 2 mg/ml trypsin (11 418 025 001; Roche) dissolved in 1% acetic acid or subtilisin (P5380; Sigma) dissolved in 100 mM Tris-HCl, pH 8.0, was added to 120 µl purified Zas1 (1.4 µg/ml in dialysis buffer) and incubated at 23°C. At the indicated time points, 15 µl of the reaction was stopped by addition of 5 µl of 5× SDS-PAGE loading buffer-PMSF (250 mM Tris-HCl, pH 6.8, 5% SDS, 50% [vol/vol] glycerol, 0.5% bromophenol blue, 100 mM DTT, and 10 µM PMSF) and incubation for 5 min at 95°C. Samples were separated by SDS-PAGE gel and Coomassie stained, and corresponding bands were cut from the gels. N and C termini of the respective protein fragments were determined by in-gel acid hydrolysis and subsequent mass spectrometry. For analysis of proteolysis fragments by SEC, the subtilisin reaction was stopped after 2 min by addition of PMSF to a final concentration of 1 mM. Fragments were separated on Superdex 200 Increase 3.2/200 column on an Äkta Ettan system (GE Healthcare) and analyzed on SDS-PAGE.

### Cnd1 protein expression and purification for polyclonal antibody production

Expression of *S. pombe* His_6_–Cnd1 was induced for 18 h from pET-MCN vectors (Romier et al., 2006) in *Escherichia coli* Rosetta (DE3) pLysS (Merck) grown at 18°C in 8 liter Terrific Broth (TB) medium. Cells were lysed by sonication at 4°C in lysis buffer (50 mM Tris-HCl, pH 7.5, 500 mM NaCl, 20 mM imidazole, and 5 mM β-mercaptoethanol containing cOmplete protease inhibitor cocktail tablets without EDTA). The lysate was cleared by centrifugation at 45,000 *g* (maximum) and incubated for 2.5 h with Ni-Sepharose 6FF (GE Healthcare) beads at 4°C. The beads were washed with 30–40 column volumes (CV) of lysis buffer, and proteins were eluted in 5–7 CV elution buffer (lysis buffer plus 300 mM imidazole). The His_6_-tag was cleaved by addition of 500 mg HRV-3C protease during overnight dialysis in 2 liter dialysis buffer (25 mM Tris-HCl, pH 7.5, 200 mM NaCl, and 1 mM DTT) at 4°C. The dialyzed protein was first diluted to a final salt concentration of 150 mM NaCl with low-salt buffer (25 mM Tris-HCl, pH 7.5, 100 mM NaCl, and 1 mM DTT) and loaded onto a 6-ml Resource Q (GE Healthcare) anion exchange column. The column was washed with 3–5 CV low-salt buffer, and proteins were eluted in a 60-ml linear salt gradient (0.1–1.0 M NaCl). Cnd1-containing peak fractions were identified by SDS-PAGE and Coomassie staining, pooled, and loaded onto a Superdex 200 26/60 column (GE Healthcare) equilibrated in SEC buffer (25 mM Tris–HCl, pH 7.5, 500 mM NaCl, and 1 mM DTT). Cnd1-containing peak fractions were identified by SDS-PAGE and Coomassie staining, pooled, and finally concentrated by ultrafiltration (Vivaspin 30,000 MWCO; Sartorius). Complete cleavage of the His_6_-tag was tested by SDS-PAGE and Western blotting using the mouse penta-His antibody (34660; Qiagen). The purified Cnd1 protein was injected in five 100-µl (1 mg/ml) boosts for rabbit immunization at the EMBL Laboratory Animal Resources/Rabbit SPF facility.

### Bioinformatics tools

The BLAST (Altschul et al., 1997) server at the EBI (https://www.ebi.ac.uk/Tools/sss/ncbiblast/) was used for routine retrieval of homologous sequences from the UniProt protein sequence database (UniProt Consortium, 2015). Multiple sequence alignments were viewed and edited using Jalview (Waterhouse et al., 2009). Sequences were submitted via the Jalview Web Services menu for alignment by Clustal Omega (Sievers et al., 2011) using the JABAWS server (http://www.compbio.dundee.ac.uk/jabaws/) hosted at University of Dundee, Scotland (Troshin et al., 2011). For secondary structure prediction, alignments were submitted to the Jpred server (Drozdetskiy et al., 2015) via the Jalview web services menu. IUPred was used for native disorder plots (Dosztányi et al., 2005). Two protein domain databases, Pfam (Finn et al., 2016) and InterPro (Finn et al., 2017), provided information about the structural modules present in Gal4- and Zas1-like protein families as well as the taxonomic ranges in which these occurred.

To discover extremely divergent homologues, more sensitive sequence searches of the UniProt database (UniProt Consortium, 2015) were performed, which used HMMs based on sequence alignments as the query. These were *hmmsearch* (https://www.ebi.ac.uk/Tools/hmmer/search/hmmsearch) from the HMMER package (Finn et al., 2015) and HHpred (https://toolkit.tuebingen.mpg.de/#/tools/hhpred; Alva et al., 2016). The latter compares a query HMM against a database of HMMs for maximal sensitivity.

To query structure databases by threading, the Phyre2 server (http://www.sbg.bio.ic.ac.uk/~phyre2/) was used (Kelley et al., 2015). Phyre2 threads an HHpred query against structures from the PDB protein data bank (Burley et al., 2017). Note that Phyre2 uses part of the HHPred software to prepare the HMM for structural threading; hence, the results obtained from the two searches are not fully independent. For structure similarity queries, the Dali structure similarity server (http://ekhidna.biocenter.helsinki.fi/dali_server/) was used (Holm and Laakso, 2016).

### Online supplemental material

Fig. S1 shows validation of Zas1 localization and ChIP-seq experiments, as well as condensin complex purification. Fig. S2 shows analyses of additional Zas1 target genes. Table S1 lists yeast genotypes. Table S2 lists sequences of primers and Zas1-binding DNA sites. The Supplemental Alignment File includes detailed sequence alignments used for the discovery of the TAD motif and the CHD.

## Supplementary Material

Supplemental Materials (PDF)

Video 1

Video 2

Tables S1 and S2 (ZIP)

Data S1 (TXT)

## References

[bib1] Adachi, Y., M. Luke, and U.K. Laemmli. 1991. Chromosome assembly in vitro: Topoisomerase II is required for condensation. Cell. 64:137–148. 10.1016/0092-8674(91)90215-K 1846085

[bib2] Afgan, E., D. Baker, M. van den Beek, D. Blankenberg, D. Bouvier, M. Čech, J. Chilton, D. Clements, N. Coraor, C. Eberhard, 2016. The Galaxy platform for accessible, reproducible and collaborative biomedical analyses: 2016 update. Nucleic Acids Res. 44(W1):W3–W10. 10.1093/nar/gkw343 27137889 PMC4987906

[bib3] Altschul, S.F., T.L. Madden, A.A. Schäffer, J. Zhang, Z. Zhang, W. Miller, and D.J. Lipman. 1997. Gapped BLAST and PSI-BLAST: A new generation of protein database search programs. Nucleic Acids Res. 25:3389–3402. 10.1093/nar/25.17.3389 9254694 PMC146917

[bib4] Alva, V., S.-Z. Nam, J. Söding, and A.N. Lupas. 2016. The MPI bioinformatics Toolkit as an integrative platform for advanced protein sequence and structure analysis. Nucleic Acids Res. 44:W410–W415. 10.1093/nar/gkw348 27131380 PMC4987908

[bib5] Anderson, D.E., A. Losada, H.P. Erickson, and T. Hirano. 2002. Condensin and cohesin display different arm conformations with characteristic hinge angles. J. Cell Biol. 156:419–424. 10.1083/jcb.200111002 11815634 PMC2173330

[bib6] Bähler, J., J.Q. Wu, M.S. Longtine, N.G. Shah, A. McKenzie III, A.B. Steever, A. Wach, P. Philippsen, and J.R. Pringle. 1998. Heterologous modules for efficient and versatile PCR-based gene targeting in Schizosaccharomyces pombe. Yeast. 14:943–951. 10.1002/(SICI)1097-0061(199807)14:10<943::AID-YEA292>3.0.CO;2-Y 9717240

[bib7] Bailey, T.L., and C. Elkan. 1994. Fitting a mixture model by expectation maximization to discover motifs in biopolymers. Proc. Int. Conf. Intell. Syst. Mol. Biol. 2:28–36. 7584402

[bib8] Bates, D.M., and J.M. Chambers. 1992. Nonlinear Models. In Statistical Models. S.J.M. Chambers, and T.J. Hastie, editors. Chapman and Hall, London. 421–453.

[bib9] Bauer, C.R., T.A. Hartl, and G. Bosco. 2012. Condensin II promotes the formation of chromosome territories by inducing axial compaction of polyploid interphase chromosomes. PLoS Genet. 8:e1002873. 10.1371/journal.pgen.1002873 22956908 PMC3431300

[bib10] Bazile, F., J. St-Pierre, and D. D’Amours. 2010. Three-step model for condensin activation during mitotic chromosome condensation. Cell Cycle. 9:3243–3255. 10.4161/cc.9.16.12620 20703077

[bib11] Bellizzi, J.J. III, P.K. Sorger, and S.C. Harrison. 2007. Crystal structure of the yeast inner kinetochore subunit Cep3p. Structure. 15:1422–1430. 10.1016/j.str.2007.09.008 17997968 PMC2288795

[bib12] Bitton, D.A., F. Schubert, S. Dey, M. Okoniewski, G.C. Smith, S. Khadayate, V. Pancaldi, V. Wood, and J. Bähler. 2015. AnGeLi: A tool for the analysis of gene lists from fission yeast. Front. Genet. 6:330. 10.3389/fgene.2015.00330 26635866 PMC4644808

[bib13] Burley, S.K., H.M. Berman, G.J. Kleywegt, J.L. Markley, H. Nakamura, and S. Velankar. 2017. Protein Data Bank (PDB): The single global macromolecular structure archive. Methods Mol. Biol. 1607:627–641. 10.1007/978-1-4939-7000-1_26 28573592 PMC5823500

[bib14] Carpy, A., K. Krug, S. Graf, A. Koch, S. Popic, S. Hauf, and B. Macek. 2014. Absolute proteome and phosphoproteome dynamics during the cell cycle of Schizosaccharomyces pombe (fission yeast). Mol. Cell. Proteomics. 13:1925–1936. 10.1074/mcp.M113.035824 24763107 PMC4125727

[bib15] Corsaro, D., J. Walochnik, D. Venditti, K.-D. Müller, B. Hauröder, and R. Michel. 2014. Rediscovery of Nucleophaga amoebae, a novel member of the Rozellomycota. Parasitol. Res. 113:4491–4498. 10.1007/s00436-014-4138-8 25258042

[bib16] Cuylen, S., J. Metz, and C.H. Haering. 2011. Condensin structures chromosomal DNA through topological links. Nat. Struct. Mol. Biol. 18:894–901. 10.1038/nsmb.2087 21765419

[bib17] Cuylen, S., J. Metz, A. Hruby, and C.H. Haering. 2013. Entrapment of chromosomes by condensin rings prevents their breakage during cytokinesis. Dev. Cell. 27:469–478. 10.1016/j.devcel.2013.10.018 24286828

[bib18] Dosztányi, Z., V. Csizmok, P. Tompa, and I. Simon. 2005. IUPred: Web server for the prediction of intrinsically unstructured regions of proteins based on estimated energy content. Bioinformatics. 21:3433–3434. 10.1093/bioinformatics/bti541 15955779

[bib19] Doughty, T.W., H.E. Arsenault, and J.A. Benanti. 2016. Levels of Ycg1 limit condensin function during the cell cycle. PLoS Genet. 12:e1006216. 10.1371/journal.pgen.1006216 27463097 PMC4963108

[bib20] Drozdetskiy, A., C. Cole, J. Procter, and G.J. Barton. 2015. JPred4: A protein secondary structure prediction server. Nucleic Acids Res. 43(W1):W389–W394. 10.1093/nar/gkv332 25883141 PMC4489285

[bib21] Feng, J., T. Liu, and Y. Zhang. 2011. Using MACS to identify peaks from ChIP-Seq data. Curr. Protoc. Bioinformatics Chapter 2:Unit 2.14–2.14.14. doi:. 10.1002/0471250953.bi0214s34 PMC3120977 21633945

[bib22] Finn, R.D., J. Clements, W. Arndt, B.L. Miller, T.J. Wheeler, F. Schreiber, A. Bateman, and S.R. Eddy. 2015. HMMER web server: 2015 update. Nucleic Acids Res. 43(W1):W30–W38. 10.1093/nar/gkv397 25943547 PMC4489315

[bib23] Finn, R.D., P. Coggill, R.Y. Eberhardt, S.R. Eddy, J. Mistry, A.L. Mitchell, S.C. Potter, M. Punta, M. Qureshi, A. Sangrador-Vegas, 2016. The Pfam protein families database: Towards a more sustainable future. Nucleic Acids Res. 44(D1):D279–D285. 10.1093/nar/gkv1344 26673716 PMC4702930

[bib24] Finn, R.D., T.K. Attwood, P.C. Babbitt, A. Bateman, P. Bork, A.J. Bridge, H.-Y. Chang, Z. Dosztányi, S. El-Gebali, M. Fraser, 2017. InterPro in 2017: Beyond protein family and domain annotations. Nucleic Acids Res. 45(D1):D190–D199. 10.1093/nar/gkw1107 27899635 PMC5210578

[bib25] Flemming, W. 1882. Zellsubstanz, Kern und Zelltheilung. Leipzig, Germany, Vogel.

[bib26] Ganji, M., I.A. Shaltiel, S. Bisht, E. Kim, A. Kalichava, C.H. Haering, and C. Dekker. 2018. Real-time imaging of DNA loop extrusion by condensin. Science. 360:102–105. 10.1126/science.aar7831 29472443 PMC6329450

[bib27] Gerlich, D., T. Hirota, B. Koch, J.-M. Peters, and J. Ellenberg. 2006. Condensin I stabilizes chromosomes mechanically through a dynamic interaction in live cells. Curr. Biol. 16:333–344. 10.1016/j.cub.2005.12.040 16488867

[bib28] Gibcus, J.H., K. Samejima, A. Goloborodko, I. Samejima, N. Naumova, J. Nuebler, M.T. Kanemaki, L. Xie, J.R. Paulson, W.C. Earnshaw, 2018. A pathway for mitotic chromosome formation. Science. 359:6135. 10.1126/science.aao6135 PMC5924687 29348367

[bib29] Gibson, T.J., and M. Kumar. 2016. Hunting for cis-regulatory elements in proteins. Cell Syst. 2:68–70. 10.1016/j.cels.2016.02.011 27135160

[bib30] Grallert, A., C. Beuter, R.A. Craven, S. Bagley, D. Wilks, U. Fleig, and I.M. Hagan. 2006. S. pombe CLASP needs dynein, not EB1 or CLIP170, to induce microtubule instability and slows polymerization rates at cell tips in a dynein-dependent manner. Genes Dev. 20:2421–2436. 10.1101/gad.381306 16951255 PMC1560416

[bib31] Helmlinger, D., S. Marguerat, J. Villén, S.P. Gygi, J. Bähler, and F. Winston. 2008. The S. pombe SAGA complex controls the switch from proliferation to sexual differentiation through the opposing roles of its subunits Gcn5 and Spt8. Genes Dev. 22:3184–3195. 10.1101/gad.1719908 19056896 PMC2593614

[bib32] Hirano, T., R. Kobayashi, and M. Hirano. 1997. Condensins, chromosome condensation protein complexes containing XCAP-C, XCAP-E and a Xenopus homolog of the Drosophila Barren protein. Cell. 89:511–521. 10.1016/S0092-8674(00)80233-0 9160743

[bib33] Hirota, T., D. Gerlich, B. Koch, J. Ellenberg, and J.-M. Peters. 2004. Distinct functions of condensin I and II in mitotic chromosome assembly. J. Cell Sci. 117:6435–6445. 10.1242/jcs.01604 15572404

[bib34] Holm, L., and L.M. Laakso. 2016. Dali server update. Nucleic Acids Res. 44(W1):W351–W355. 10.1093/nar/gkw357 27131377 PMC4987910

[bib35] Houlard, M., J. Godwin, J. Metson, J. Lee, T. Hirano, and K. Nasmyth. 2015. Condensin confers the longitudinal rigidity of chromosomes. Nat. Cell Biol. 17:771–781. 10.1038/ncb3167 25961503 PMC5207317

[bib36] Kelley, L.A., S. Mezulis, C.M. Yates, M.N. Wass, and M.J.E. Sternberg. 2015. The Phyre2 web portal for protein modeling, prediction and analysis. Nat. Protoc. 10:845–858. 10.1038/nprot.2015.053 25950237 PMC5298202

[bib37] Kim, D.-U., J. Hayles, D. Kim, V. Wood, H.-O. Park, M. Won, H.-S. Yoo, T. Duhig, M. Nam, G. Palmer, 2010. Analysis of a genome-wide set of gene deletions in the fission yeast Schizosaccharomyces pombe. Nat. Biotechnol. 28:617–623. 10.1038/nbt.1628 20473289 PMC3962850

[bib38] Kschonsak, M., F. Merkel, S. Bisht, J. Metz, V. Rybin, M. Hassler, and C.H. Haering. 2017. Structural basis for a safety-belt mechanism that anchors condensin to chromosomes. Cell. 171:588–600. 10.1016/j.cell.2017.09.008 28988770 PMC5651216

[bib39] Landt, S.G., G.K. Marinov, A. Kundaje, P. Kheradpour, F. Pauli, S. Batzoglou, B.E. Bernstein, P. Bickel, J.B. Brown, P. Cayting, 2012. ChIP-seq guidelines and practices of the ENCODE and modENCODE consortia. Genome Res. 22:1813–1831. 10.1101/gr.136184.111 22955991 PMC3431496

[bib40] Langmead, B., and S.L. Salzberg. 2012. Fast gapped-read alignment with Bowtie 2. Nat. Methods. 9:357–359. 10.1038/nmeth.1923 22388286 PMC3322381

[bib41] Lee, C., J.H. Chang, H.S. Lee, and Y. Cho. 2002. Structural basis for the recognition of the E2F transactivation domain by the retinoblastoma tumor suppressor. Genes Dev. 16:3199–3212. 10.1101/gad.1046102 12502741 PMC187509

[bib42] Lee, J., S. Ogushi, M. Saitou, and T. Hirano. 2011. Condensin I and II are essential for construction of bivalent chromosomes in mouse oocytes. Mol. Biol. Cell. 22:3465–3477. 10.1091/mbc.E11-05-0423 21795393 PMC3172270

[bib43] Liu, W., B. Liang, H. Liu, Y. Huang, X. Yin, F. Zhou, X. Yu, Q. Feng, E. Li, Z. Zou, and L. Wu. 2017. Overexpression of non-SMC condensin I complex subunit G serves as a promising prognostic marker and therapeutic target for hepatocellular carcinoma. Int. J. Mol. Med. 40:731–738. 10.3892/ijmm.2017.3079 28737823 PMC5547945

[bib44] Longtine, M.S., A. McKenzie III, D.J. Demarini, N.G. Shah, A. Wach, A. Brachat, P. Philippsen, and J.R. Pringle. 1998. Additional modules for versatile and economical PCR-based gene deletion and modification in Saccharomyces cerevisiae. Yeast. 14:953–961. 10.1002/(SICI)1097-0061(199807)14:10<953::AID-YEA293>3.0.CO;2-U 9717241

[bib45] Magalska, A., A.K. Schellhaus, D. Moreno-Andrés, F. Zanini, A. Schooley, R. Sachdev, H. Schwarz, J. Madlung, and W. Antonin. 2014. RuvB-like ATPases function in chromatin decondensation at the end of mitosis. Dev. Cell. 31:305–318. 10.1016/j.devcel.2014.09.001 25443297

[bib46] McDowall, M.D., M.A. Harris, A. Lock, K. Rutherford, D.M. Staines, J. Bähler, P.J. Kersey, S.G. Oliver, and V. Wood. 2015. PomBase 2015: Updates to the fission yeast database. Nucleic Acids Res. 43(D1):D656–D661. 10.1093/nar/gku1040 25361970 PMC4383888

[bib47] Moser, S.C., and J.R. Swedlow. 2011. How to be a mitotic chromosome. Chromosome Res. 19:307–319. 10.1007/s10577-011-9198-3 21461697 PMC3078314

[bib48] Murray, J.M., A.T. Watson, and A.M. Carr. 2016 a. Transformation of Schizosaccharomyces pombe: Lithium acetate/ dimethyl sulfoxide procedure. Cold Spring Harb. Protoc. 10.1101/pdb.prot090969 27037075

[bib49] Murray, J.M., A.T. Watson, and A.M. Carr. 2016 b. Extraction of chromosomal DNA from Schizosaccharomyces pombe. Cold Spring Harb. Protoc. 2016:pdb.prot090985. 10.1101/pdb.prot090985 27140918

[bib50] Naumova, N., M. Imakaev, G. Fudenberg, Y. Zhan, B.R. Lajoie, L.A. Mirny, and J. Dekker. 2013. Organization of the mitotic chromosome. Science. 342:948–953. 10.1126/science.1236083 24200812 PMC4040465

[bib51] Ohta, S., J.-C. Bukowski-Wills, L. Sanchez-Pulido, F.L. Alves, L. Wood, Z.A. Chen, M. Platani, L. Fischer, D.F. Hudson, C.P. Ponting, 2010. The protein composition of mitotic chromosomes determined using multiclassifier combinatorial proteomics. Cell. 142:810–821. 10.1016/j.cell.2010.07.047 20813266 PMC2982257

[bib52] Okazaki, K., and O. Niwa. 2000. mRNAs encoding zinc finger protein isoforms are expressed by alternative splicing of an in-frame intron in fission yeast. DNA Res. 7:27–30. 10.1093/dnares/7.1.27 10718196

[bib53] Oliveira, R.A., S. Heidmann, and C.E. Sunkel. 2007. Condensin I binds chromatin early in prophase and displays a highly dynamic association with Drosophila mitotic chromosomes. Chromosoma. 116:259–274. 10.1007/s00412-007-0097-5 17318635

[bib54] Onn, I., N. Aono, M. Hirano, and T. Hirano. 2007. Reconstitution and subunit geometry of human condensin complexes. EMBO J. 26:1024–1034. 10.1038/sj.emboj.7601562 17268547 PMC1852836

[bib55] Ono, T., A. Losada, M. Hirano, M.P. Myers, A.F. Neuwald, and T. Hirano. 2003. Differential contributions of condensin I and condensin II to mitotic chromosome architecture in vertebrate cells. Cell. 115:109–121. 10.1016/S0092-8674(03)00724-4 14532007

[bib56] Pagès, H. 2017. BSgenome: Software infrastructure for efficient representation of full genomes and their SNPs. R Package version 1.46.0. https://rdrr.io/bioc/BSgenome/. Accessed April 9, 2018.

[bib57] Pavletich, N.P., and C.O. Pabo. 1991. Zinc finger-DNA recognition: Crystal structure of a Zif268-DNA complex at 2.1 A. Science. 252:809–817. 10.1126/science.2028256 2028256

[bib58] Peng, X., R.K.M. Karuturi, L.D. Miller, K. Lin, Y. Jia, P. Kondu, L. Wang, L.-S. Wong, E.T. Liu, M.K. Balasubramanian, and J. Liu. 2005. Identification of cell cycle-regulated genes in fission yeast. Mol. Biol. Cell. 16:1026–1042. 10.1091/mbc.E04-04-0299 15616197 PMC551471

[bib59] Persikov, A.V., and M. Singh. 2014. De novo prediction of DNA-binding specificities for Cys2His2 zinc finger proteins. Nucleic Acids Res. 42:97–108. 10.1093/nar/gkt890 24097433 PMC3874201

[bib60] Petrova, B., S. Dehler, T. Kruitwagen, J.-K. Hériché, K. Miura, and C.H. Haering. 2013. Quantitative analysis of chromosome condensation in fission yeast. Mol. Cell. Biol. 33:984–998. 10.1128/MCB.01400-12 23263988 PMC3623070

[bib61] Pfaffl, M.W. 2001. A new mathematical model for relative quantification in real-time RT-PCR. Nucleic Acids Res. 29:e45. 10.1093/nar/29.9.e45 11328886 PMC55695

[bib62] Piazza, I., C.H. Haering, and A. Rutkowska. 2013. Condensin: Crafting the chromosome landscape. Chromosoma. 122:175–190. 10.1007/s00412-013-0405-1 23546018

[bib63] Piazza, I., A. Rutkowska, A. Ori, M. Walczak, J. Metz, V. Pelechano, M. Beck, and C.H. Haering. 2014. Association of condensin with chromosomes depends on DNA binding by its HEAT-repeat subunits. Nat. Struct. Mol. Biol. 21:560–568. 10.1038/nsmb.2831 24837193

[bib64] Piskacek, M., M. Havelka, M. Rezacova, and A. Knight. 2016. The 9aaTAD transactivation domains: From Gal4 to p53. PLoS One. 11:e0162842. 10.1371/journal.pone.0162842 27618436 PMC5019370

[bib65] Piskadlo, E., A. Tavares, and R.A. Oliveira. 2017. Metaphase chromosome structure is dynamically maintained by condensin I-directed DNA (de)catenation. eLife. 6:e26120. 10.7554/eLife.26120 28477406 PMC5451211

[bib66] Purvis, A., and M.R. Singleton. 2008. Insights into kinetochore-DNA interactions from the structure of Cep3Delta. EMBO Rep. 9:56–62. 10.1038/sj.embor.7401139 18064045 PMC2246632

[bib67] Rhind, N., Z. Chen, M. Yassour, D.A. Thompson, B.J. Haas, N. Habib, I. Wapinski, S. Roy, M.F. Lin, D.I. Heiman, 2011. Comparative functional genomics of the fission yeasts. Science. 332:930–936. 10.1126/science.1203357 21511999 PMC3131103

[bib68] Romier, C., M. Ben Jelloul, S. Albeck, G. Buchwald, D. Busso, P.H.N. Celie, E. Christodoulou, V. De Marco, S. van Gerwen, P. Knipscheer, 2006. Co-expression of protein complexes in prokaryotic and eukaryotic hosts: Experimental procedures, database tracking and case studies. Acta Crystallogr. D Biol. Crystallogr. 62:1232–1242. 10.1107/S0907444906031003 17001100

[bib69] Rustici, G., J. Mata, K. Kivinen, P. Lió, C.J. Penkett, G. Burns, J. Hayles, A. Brazma, P. Nurse, and J. Bähler. 2004. Periodic gene expression program of the fission yeast cell cycle. Nat. Genet. 36:809–817. 10.1038/ng1377 15195092

[bib70] Sawin, K.E., and P. Nurse. 1996. Identification of fission yeast nuclear markers using random polypeptide fusions with green fluorescent protein. Proc. Natl. Acad. Sci. USA. 93:15146–15151. 10.1073/pnas.93.26.15146 8986778 PMC26371

[bib71] Schiklenk, C., B. Petrova, and C.H. Haering. 2017. A protocol for measuring mitotic chromosome condensation quantitatively in fission yeast cells. Methods Mol. Biol. 1515:245–255. 10.1007/978-1-4939-6545-8_15 27797084

[bib72] Sen, N., J. Leonard, R. Torres, J. García-Luis, G. Palou-Marin, and L. Aragón. 2016. Physical proximity of sister chromatids promotes Top2-dependent intertwining. Mol. Cell. 64:134–147. 10.1016/j.molcel.2016.09.007 27716481 PMC5065527

[bib73] Shimanuki, M., L. Uehara, T. Pluskal, T. Yoshida, A. Kokubu, Y. Kawasaki, and M. Yanagida. 2013. Klf1, a C2H2 zinc finger-transcription factor, is required for cell wall maintenance during long-term quiescence in differentiated G0 phase. PLoS One. 8:e78545. 10.1371/journal.pone.0078545 24167631 PMC3805531

[bib74] Shintomi, K., T.S. Takahashi, and T. Hirano. 2015. Reconstitution of mitotic chromatids with a minimum set of purified factors. Nat. Cell Biol. 17:1014–1023. 10.1038/ncb3187 26075356

[bib75] Shintomi, K., F. Inoue, H. Watanabe, K. Ohsumi, M. Ohsugi, and T. Hirano. 2017. Mitotic chromosome assembly despite nucleosome depletion in *Xenopus* egg extracts. Science. 356:1284–1287. 10.1126/science.aam9702 28522692

[bib76] Sievers, F., A. Wilm, D. Dineen, T.J. Gibson, K. Karplus, W. Li, R. Lopez, H. McWilliam, M. Remmert, J. Söding, 2011. Fast, scalable generation of high-quality protein multiple sequence alignments using Clustal Omega. Mol. Syst. Biol. 7:539. 10.1038/msb.2011.75 21988835 PMC3261699

[bib77] Staby, L., C. O’Shea, M. Willemoës, F. Theisen, B.B. Kragelund, and K. Skriver. 2017. Eukaryotic transcription factors: Paradigms of protein intrinsic disorder. Biochem. J. 474:2509–2532. 10.1042/BCJ20160631 28701416

[bib78] Sutani, T., T. Yuasa, T. Tomonaga, N. Dohmae, K. Takio, and M. Yanagida. 1999. Fission yeast condensin complex: Essential roles of non-SMC subunits for condensation and Cdc2 phosphorylation of Cut3/SMC4. Genes Dev. 13:2271–2283. 10.1101/gad.13.17.2271 10485849 PMC316991

[bib79] Sutani, T., T. Sakata, R. Nakato, K. Masuda, M. Ishibashi, D. Yamashita, Y. Suzuki, T. Hirano, M. Bando, and K. Shirahige. 2015. Condensin targets and reduces unwound DNA structures associated with transcription in mitotic chromosome condensation. Nat. Commun. 6:7815. 10.1038/ncomms8815 26204128 PMC4525155

[bib80] Terakawa, T., S. Bisht, J.M. Eeftens, C. Dekker, C.H. Haering, and E.C. Greene. 2017. The condensin complex is a mechanochemical motor that translocates along DNA. Science. 358:672–676. 10.1126/science.aan6516 28882993 PMC5862036

[bib81] Troshin, P.V., J.B. Procter, and G.J. Barton. 2011. Java bioinformatics analysis web services for multiple sequence alignment—JABAWS:MSA. Bioinformatics. 27:2001–2002. 10.1093/bioinformatics/btr304 21593132 PMC3129525

[bib82] Uemura, T., H. Ohkura, Y. Adachi, K. Morino, K. Shiozaki, and M. Yanagida. 1987. DNA topoisomerase II is required for condensation and separation of mitotic chromosomes in S. pombe. Cell. 50:917–925. 10.1016/0092-8674(87)90518-6 3040264

[bib83] UniProt Consortium. 2015. UniProt: A hub for protein information. Nucleic Acids Res. 43(D1):D204–D212. 10.1093/nar/gku989 25348405 PMC4384041

[bib84] Wach, A., A. Brachat, R. Pöhlmann, and P. Philippsen. 1994. New heterologous modules for classical or PCR-based gene disruptions in Saccharomyces cerevisiae. Yeast. 10:1793–1808. 10.1002/yea.320101310 7747518

[bib85] Waterhouse, A.M., J.B. Procter, D.M.A. Martin, M. Clamp, and G.J. Barton. 2009. Jalview Version 2—A multiple sequence alignment editor and analysis workbench. Bioinformatics. 25:1189–1191. 10.1093/bioinformatics/btp033 19151095 PMC2672624

[bib86] Wickham, H. 2009. ggplot2: Elegant Graphics for Data Analysis. Springer, New York.

[bib87] Wickham, H., R. Francois, L. Henry, and K. Müller. 2018. The Dplyr Package. http://dplyr.tidyverse.org/. Accessed April 9, 2018.

[bib88] Yang, J., X. Chen, A. McDermaid, and Q. Ma. 2017. DMINDA 2.0: Integrated and systematic views of regulatory DNA motif identification and analyses. Bioinformatics. 33:2586–2588. 10.1093/bioinformatics/btx223 28419194

[bib89] Zhan, P., G.-M. Xi, B. Zhang, Y. Wu, H.-B. Liu, Y.-F. Liu, W.-J. Xu, Q. Zhu, F. Cai, Z.-J. Zhou, 2017. NCAPG2 promotes tumour proliferation by regulating G2/M phase and associates with poor prognosis in lung adenocarcinoma. J. Cell. Mol. Med. 21:665–676. 10.1111/jcmm.13010 27862966 PMC5345611

[bib90] Zhang, Q., R. Su, C. Shan, C. Gao, and P. Wu. 2018. Non-SMC condensin I complex, subunit G (NCAPG) is a novel mitotic gene required for hepatocellular cancer cell proliferation and migration. Oncol. Res. 26:269–276. 10.3727/096504017X15075967560980 29046167 PMC7844763

[bib91] Zhang, Y., T. Liu, C.A. Meyer, J. Eeckhoute, D.S. Johnson, B.E. Bernstein, C. Nusbaum, R.M. Myers, M. Brown, W. Li, and X.S. Liu. 2008. Model-based analysis of ChIP-Seq (MACS). Genome Biol. 9:R137. 10.1186/gb-2008-9-9-r137 18798982 PMC2592715

