# Supplementary material for: Control of mitotic chromosome condensation by the fission yeast transcription factor Zas1
Source: J Cell Biol. 2018 Jul 2;217(7):2383–401. doi: 10.1083/jcb.201711097 (PMC6028546; doi:10.1083/jcb.201711097)
Supplement: Supplemental Materials (PDF) [file JCB_201711097_sm.pdf]

JCB

Supplemental material

Schiklenk et al., https://doi.org/10.1083/jcb.201711097

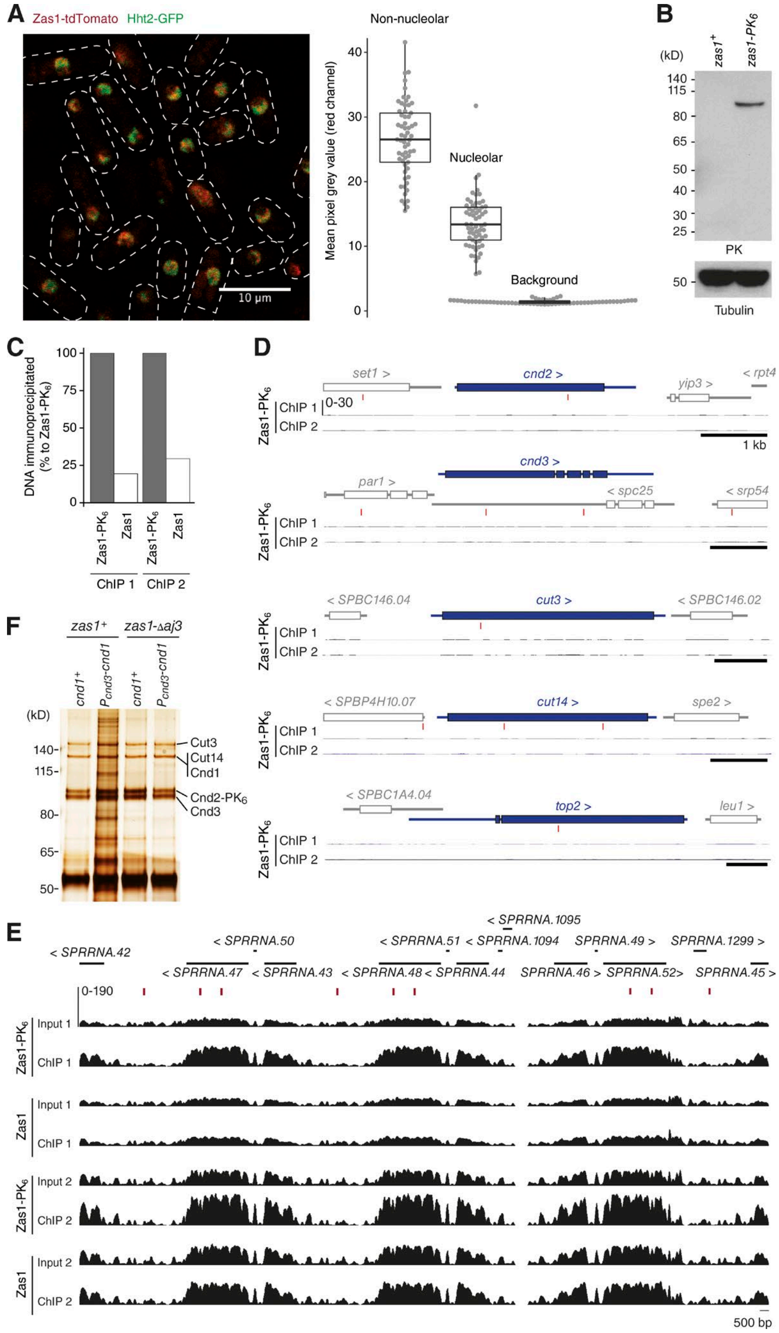

Figure S1. **Characterization of Zas1 binding to chromosomes.** **(A)** Confocal section of asynchronous cells that coexpress Hht2-GFP and Zas1-tdTomato (strain C5182). The graph shows the mean Zas1-tdTomato fluorescent signal intensities in nonnucleolar, nucleolar, and background regions (bar, median; boxes, upper and lower quartiles; whiskers, minima and maxima with 1.5 interquartile range outlier rule; *n* = 61 cells). **(B)** Immunoblot of whole-cell extracts from yeast strains (C28, C4120) expressing untagged or PK_6-tagged versions of Zas1. Immunoblotting against α-tubulin serves as loading control. **(C)** Total DNA levels coimmunoprecipitated with Zas1 in relation to Zas1-PK_6 from strains in B in two independent ChIP experiments determined by fluorometric quantitation (Qubit dsDNA HS assay). **(D)** Sequencing read profiles at the *cnd2*, *cnd3*, *cut3*, *cut14* condensin, and *top2* topo II gene loci of ChIP samples from two independent experiments with strains in B. Vertical red lines indicate positions of Zas1-UASs; black horizontal bar indicates 1 kb. **(E)** Sequencing read profiles mapped to three rDNA repeat regions of a representative ChIP experiment with strains in B. Red lines indicate the positions of Zas1-UAS motifs. **(F)** Condensin complexes were immunoprecipitated from asynchronous cultures using an antibody against ta PK_6 epitope fused to the C terminus of Cnd2 and analyzed by SDS-PAGE and silver staining.

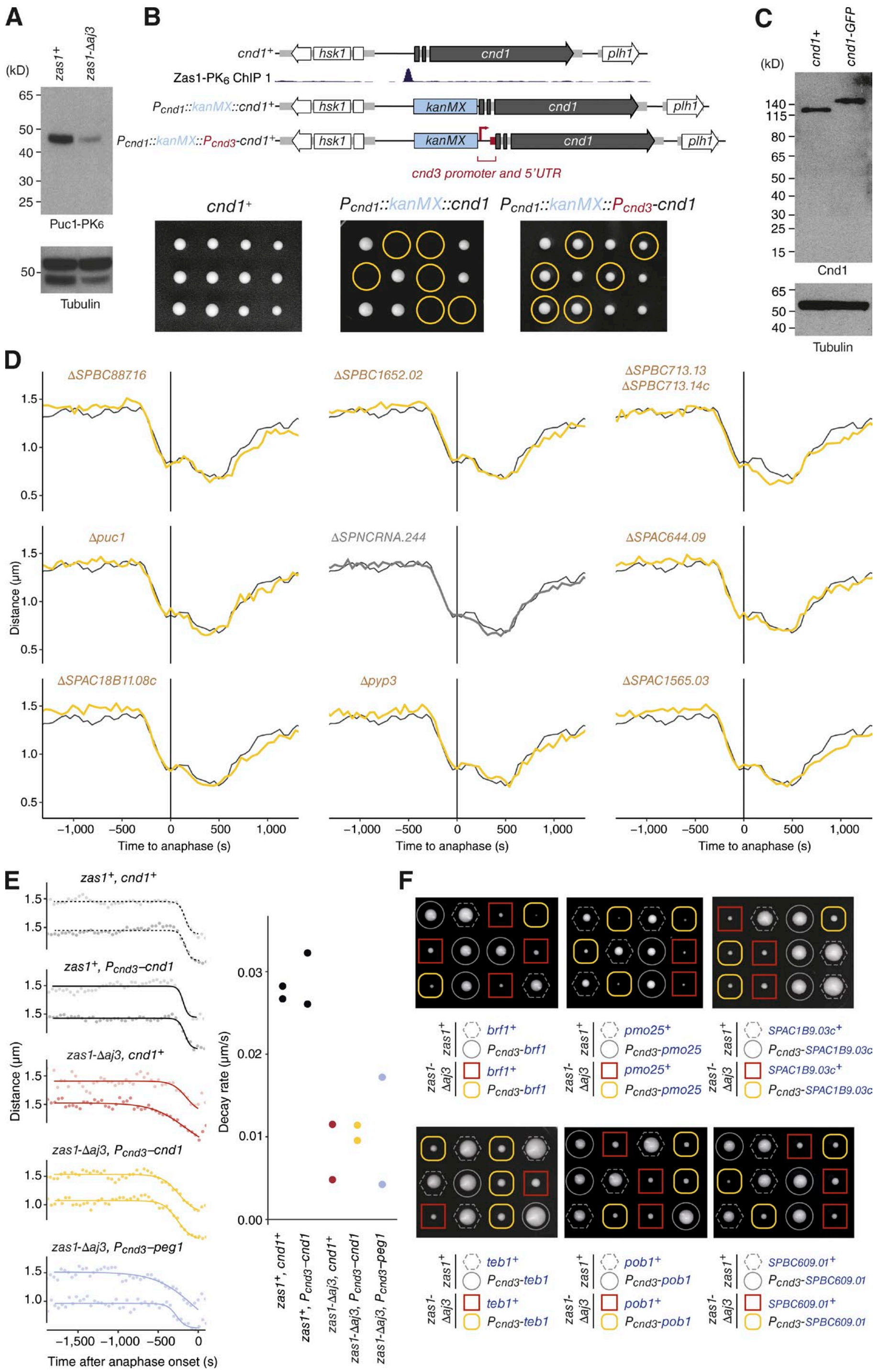

Figure S2. **Characterization of Zas1 target genes. (A)** Comparison of protein levels by immunoblotting of whole-cell extracts from asynchronous cultures of strains derived from dissection of the same *zas1*⁺/*zas1-Δaj3* tetrad (C4674) against the PK_6 tag fused to Puc1. Immunoblotting against α-tubulin serves as loading control. **(B)** Schematic of the disruption of the endogenous *cnd1* promoter by insertion of the kanMX resistance marker cassette followed by insertion of the *cnd3* promoter between the *cnd1* promoter and the start codon of the *cnd1* ORF. Tetrad analysis of heterozygous *cnd1*⁺/*cnd1*⁺, P_{cnd1}*Δ-cnd1*/*cnd1*⁺, and P_{cnd3}*-cnd1*/*cnd1*⁺ diploid fission yeast strains (C4632, C4648, C4485) after 5 d at 25°C. Circles identify the position of spores bearing the *cnd1*⁺, P_{cnd1}*Δ-cnd1*, and P_{cnd3}*-cnd1* genes, respectively. **(C)** Validation of the Cnd1 antibody by blotting of whole-cell extracts from cells expressing untagged or GFP-tagged versions of *cnd1*⁺ from their endogenous genomic loci (C28, C4660). **(D)** Comparison of condensation curves of wild-type (black, C4960, *n* = 149 cells) and *ΔSPBC887.16* (yellow, C5104, *n* = 99 cells), *ΔSPBC1652.02* (yellow, C5105, *n* = 177 cells), *ΔSPBC713.13 ΔSPBC713.14c* (yellow, C5089, *n* = 128 cells), *Δpuc1* (yellow, C5088, *n* = 124), *ΔSPNCRNA.244* (gray, C5138, *n* = 144 cells), *ΔSPAC644.09* (yellow, C5109, *n* = 163 cells), *ΔSPAC18B11.08c* (yellow, C5084, *n* = 143 cells), *Δpyp3* (yellow, C5090, *n* = 100 cells), and *ΔSPAC1565.03* (yellow, C5085, *n* = 136 cells) strains. **(E)** Sigmoid fits of condensation curves shown in Fig. 5 (E and H) from two independent experiments. The plot shows the decay rates calculated from the two fits for each experiment. **(F)** Tetrad analysis of a heterozygous *zas1-Δaj3*/*zas1*⁺, P_{cnd3}*-brf1*/*brf1*⁺ (C5094), P_{cnd3}*-SPAC1B9.03c*/*SPAC1B9.03c*⁺ (C5077), P_{cnd3}*-teb1*/*teb1*⁺ (C5078), P_{cnd3}*-pob1*/*pob*⁺ (C5093), and P_{cnd3}*-SPBC609.01*/*SPBC609.01*⁺ (C5103) diploid fission yeast strains imaged after 5 d at 25°C.

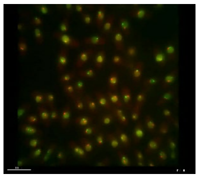

Video 1. **Principle of the chromosome condensation assay.** A z-stack video of a monolayer of *S. pombe* cells carrying red and green FROS labels on one arm of chromosome I was acquired in YE5S medium every 45 s for 1 h. 3D positions of FROS foci were tracked in cells that divided during acquisition. Bar, 10 µm.

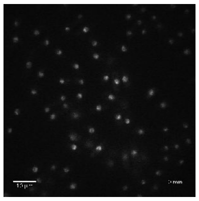

Video 2. **Zas1-tdTomato localizes to the nucleus throughout the cell cycle.** Live-cell time-lapse recording of haploid cells bearing the Zas1-tdTomato allele (C5092) in EMM2 medium. Bar, 10 µm; frame rate, 1/min.

Provided online are two tables in Excel. Table S1 lists yeast genotypes. Table S2 lists sequences of primers and Zas1-binding DNA sites.

The supplemental alignment file (TXT file) includes detailed sequence alignments used for the discovery of the TAD motif and the CHD.
